# Biological activity and structure–activity relationship of dehydrodieugenol B analogues against visceral leishmaniasis†

**DOI:** 10.1039/d3md00081h

**Published:** 2023-05-26

**Authors:** Maiara Amaral, Hannah Asiki, Claire E. Sear, Snigdha Singh, Pauline Pieper, Marius M. Haugland, Edward A. Anderson, Andre G. Tempone

**Affiliations:** a Instituto de Medicina Tropical, Faculdade de Medicina, Universidade de Sao Paulo Sao Paulo – 05403-000 Brazil; b Centre for Parasitology and Mycology, Instituto Adolfo Lutz São Paulo – 01246-000 Brazil andre.tempone@ial.sp.gov.br atempone@usp.br; c Chemistry Research Laboratory, Department of Chemistry, University of Oxford 12 Mansfield Road Oxford OX1 3TA UK edward.anderson@chem.ox.ac.uk

## Abstract

Visceral leishmaniasis is a neglected protozoan disease with high mortality. Existing treatments exhibit a number of limitations, resulting in a significant challenge for public health, especially in developing countries in which the disease is endemic. With a limited pipeline of potential drugs in clinical trials, natural products could offer an attractive source of new pharmaceutical prototypes, not least due to their high chemodiversity. In the present work, a study of anti-*L.* (*L.*) *infantum* potential was carried out for a series of 39 synthetic compounds based on the core scaffold of the neolignan dehydrodieugenol B. Of these, 14 compounds exhibited activity against intracellular amastigotes, with 50% inhibitory concentration (IC_50_) values between 3.0 and 32.7 μM. A structure–activity relationship (SAR) analysis demonstrated a requirement for polar functionalities to improve activity. Lacking mammalian cytotoxicity and presenting the highest potency against the clinically relevant form of the parasite, compound 24 emerged as the most promising, fulfilling the hit criteria for visceral leishmaniasis defined by the Drugs for Neglected Diseases *initiative* (DND*i*). This study emphasizes the potential of dehydrodieugenol B analogues as new candidates for the treatment of visceral leishmaniasis and suggests 24 to be a suitable compound for future optimization, including mechanism of action and pharmacokinetic studies.

## Abstract 2

39 synthetic compounds based on the core scaffold of the neoligan dehydrodieugenol B presented activity against intracellular amastigotes of *Leishmania*.

## Graphical abstract

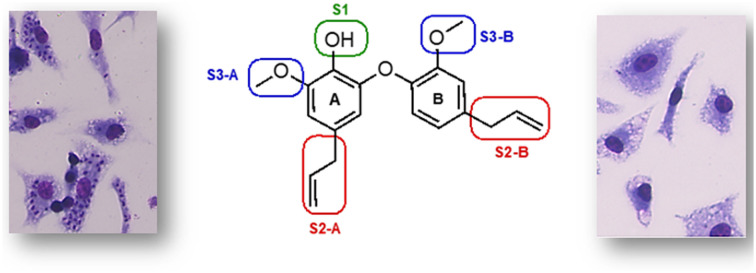

## Introduction

Leishmaniasis are neglected tropical diseases caused by parasites of the genus *Leishmania* which affect more than 12 million people worldwide, mainly in low-income populations. The disease is present in 98 countries, with approximately 1.3 million new cases per year and 350 million people at risk of infection.^1,2^ Human visceral leishmaniasis (VL) is a systemic disease which typically affects mononuclear phagocytic system cells. If treated, VL mortality rates can be as low as 10 to 20%; but if untreated, the disease is 100% fatal within two years.^3^ Although VL is the most severe form of the disease, it has received relatively little attention in terms of new treatments; a limited chemotherapeutic arsenal is available, with many current therapies eliciting adverse effects.^4^ As a result, the search for safe and accessible therapeutic alternatives is of exceptional importance.

In the search for new drugs, natural products stand out as compounds that offer enormous pharmacological potential. Newman and Cragg (2020) showed that approximately 50% of all FDA-approved drugs feature natural product scaffolds as the basis of their pharmacophores.^5^ Our previous studies described the anti-*Leishmania* (*L.*) *donovani* and anti-*Trypanosoma* (*T.*) *cruzi* activity of a small family of neolignans isolated from branches and leaves of *Nectandra leucanta* (*Lauraceae*).^6–8^ Subsequently, using dehydrodieugenol B, four new semi-synthetic compounds were prepared and their antileishmanial activity reported.^9^

**Fig. 1:**
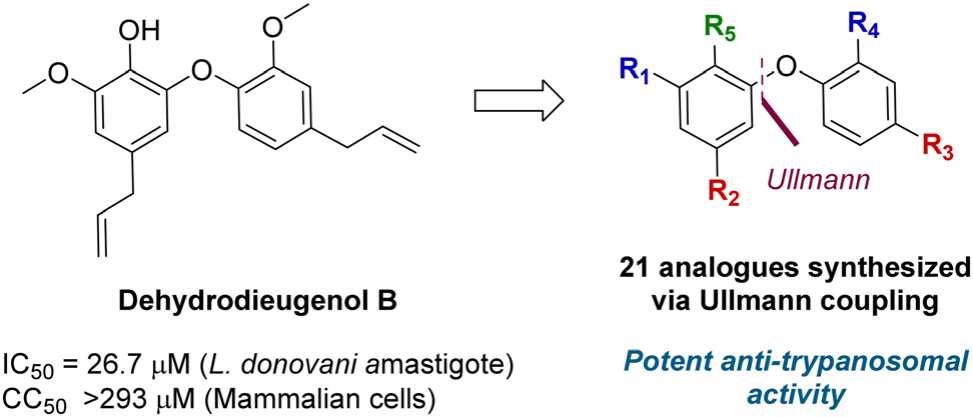
Previous work on dehydrodieugenol B synthesis and anti-parasitic activity of natural product analogues.

A total synthesis of dehydrodieugenol B was developed based on a copper-catalyzed Ullmann coupling to form the biaryl ether core (Fig. 1).^10^ This enabled site-specific modifications, and evaluation of a series of analogues against *T. cruzi*, resulting in the identification of several compounds that displayed enhanced bioactivity and selectivity against the trypomastigote form of the parasite, and meeting the Drugs for Neglected Diseases *initiative* (DND*i*) criteria for hit compounds.^11^

In this work, the bioactivity of a series of 39 analogues of dehydrodieugenol B was studied against *Leishmania* (*L.*) *infantum* intracellular amastigotes. These compounds feature a variety of structural modifications which aim to improve biological activity over the natural product, and also to obtain information about the pharmacophore. Additionally, absorption, distribution, metabolism, excretion and toxicity (ADMET) parameters of the most promising compounds were evaluated using an *in silico* platform to investigate the drug-likeness profile.

## Results and discussion

**Fig. 2:**
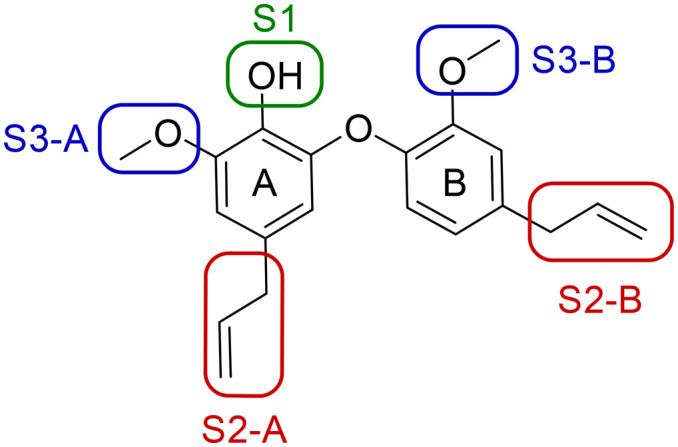
SAR strategy to explore antileishmanial activity of the dehydrodieugenol B scaffold.

A series of 39 compounds was synthesized to explore the structure–activity relationships (SAR) of this family as antileishmanial compounds. Three different approaches were employed: I) substitution on the phenol group of the A-ring (S1); II) saturation, substitution or removal of the allyl side chains (S2-A and B), and III) removal of the methoxy groups (S3-A or B) (Fig. 2). These compounds were prepared by similar chemistry to our previous work,^10^ using an Ullmann coupling to form the C–O biaryl ether bond to the A ring from appropriately functionalized precursors (see the ESI† for synthesis details).

The synthesized compounds were evaluated for their anti-*L.* (*L.*) *infantum* activity on intracellular amastigotes following 96 h treatment, with standard drug miltefosine used as a control. The 50% inhibitory concentration (IC_50_) was determined by the infection index,^12^ and mammalian toxicity (CC_50_) for the series was evaluated against NCTC cells.

We first considered modification at the S1 (phenol) position of dehydrodieugenol B (Table 1). Although the *p*-methoxybenzyl ether of the natural product (1) had shown high activity against *T. cruzi* trypomastigotes in our previous work (4.0 ± 1.4 μM), this derivative proved inactive against *L.* (*L.*) *infantum* amastigotes. The introduction of a polar group on the sidechain seems to be necessary for activity, as other modifications at this position were inactive. Having a larger p*K*_a_ is beneficial for these compounds as the more basic amines (4, 5) exhibited lower IC_50_ values than their corresponding amides (6, 7), although these four compounds also displayed moderate mammalian cytotoxicity.

**Table 1:** Anti-*Leishmania* (*L.*) *infantum* activity and mammalian cytotoxicity of the dehydrodieugenol B analogues, modification at S1

Comp.	S1	IC_50_ (μM) ± SD	CC_50_ (μM) ± SD	SI
1	OPMB	NA	>200	ND
2	OMOM	NA	>200	ND
3	OAllyl	NA	>200	ND
4	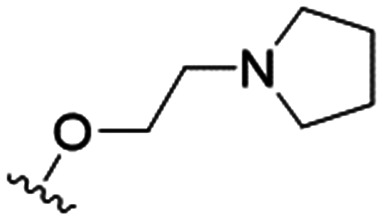	4.1 ± 1.1	24.9 ± 2.6	6
5	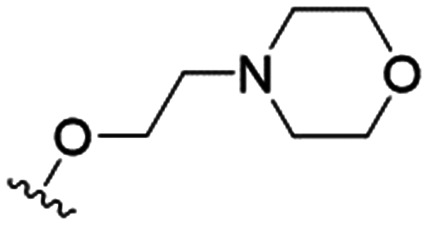	9.6 ± 3.6	63.7 ± 0.2	6.6
6	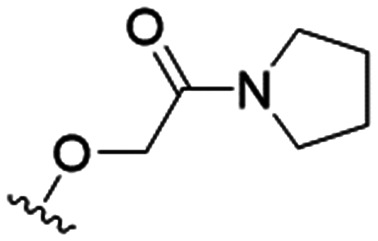	20.3 ± 5.9	52.9 ± 15.2	2.6
7	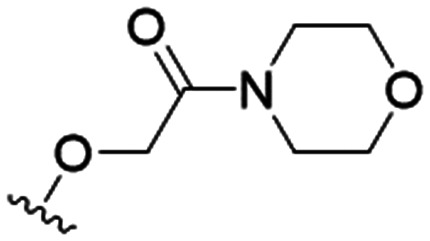	NA	111.5 ± 8.2	ND
8	H	NA	>200	ND
Milt.	—	6.5 ± 3.0	119.7 ± 4.2	18.4

Next, the effect of modifying the allyl sidechain functionalities on each of the A and B rings (positions S2) by substitution, saturation or deletion was considered (Table 2). Saturation or deletion of either of the allyl groups did not lead to antileishmanial activity in any of the analogues prepared, with the exception of moderate activity in 9 and 11. We noted a detrimental effect on the CC_50_ values in 15, 17, and 18, but this is more likely due to the presence of a free phenol in position S1 and not a result of the saturations or deletions (a result we had observed in our analogous work on *T. cruzi*); all other derivatives showed no toxicity to mammalian cells at the tested concentrations.

**Table 2:** Anti-*Leishmania* (*L.*) *infantum* activity and mammalian cytotoxicity of the dehydrodieugenol B analogues, modification at S2

Comp	S1	S2A	S2B	IC_50_ (μM) ± SD	CC_50_ (μM) ± SD	SI
9	OPMB	Allyl	H	24.2 ± 18.2	>200	>8.3
10	OPMB	H	Allyl	NA	>200	ND
11	OAcyl	Allyl	H	32.7 ± 5.0	>200	>6.1
12	OAcyl	H	Allyl	NA	>200	ND
13	OMe	Allyl	H	NA	>200	ND
14	OMe	H	Allyl	NA	>200	ND
15	OH	Allyl	H	NA	31.6 ± 11.3	ND
16	OH	H	Allyl	NA	>200	ND
17	OH	Allyl	*n*-Pr	NA	42.0 ± 3.8	ND
18	OH	*n*-Pr	Allyl	NA	14.2 ± 0.1	ND
19	OMe	Allyl	*n*-Pr	NA	>200	ND
20	OPMB	Allyl	*n*-Pr	NA	>200	ND
21	OPMB	*n*-Pr	Allyl	NA	>200	ND
22	OPMB	*n*-Pr	*n*-Pr	NA	>200	ND
23	OPMB	Allyl	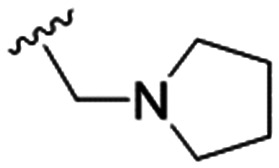	NA	6.2 ± 1.2	ND
24	OPMB	Allyl	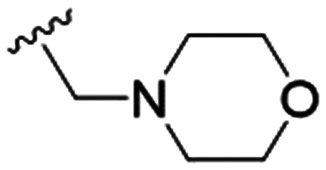	9.7 ± 2.0	>200	>20.6
25	OPMB	*n*-Pr	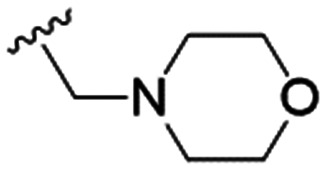	7.7 ± 0.8	74.4 ± 4.4	9.7
26	OPMB	*n*-Pr	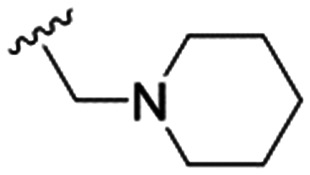	3.7 ± 0.5	14.5 ± 0.2	3.9
27	OPMB	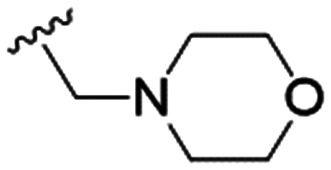	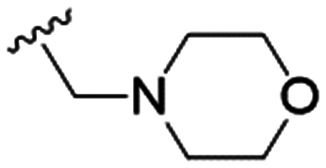	27.8 ± 7.4	74.4 ± 5.8	2.7
28	OPMB	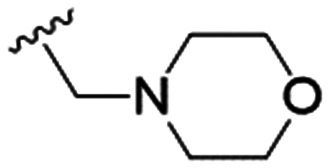	*n*-Pr	16.4 ± 4.3	>200	>12.2
29	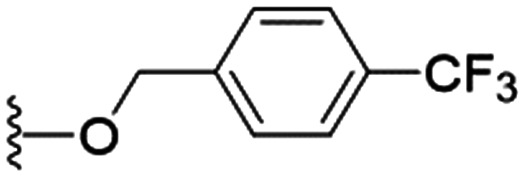	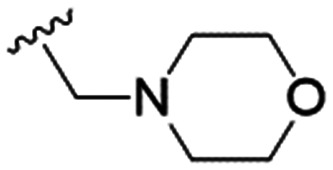	*n*-Pr	13.2 ± 2.1	>200	>15.1
30	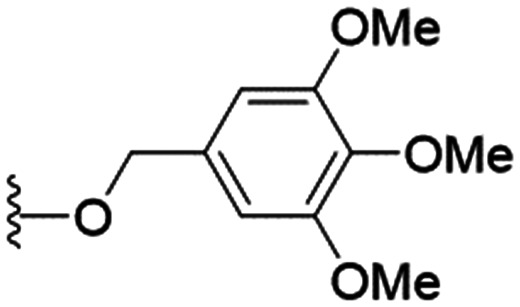	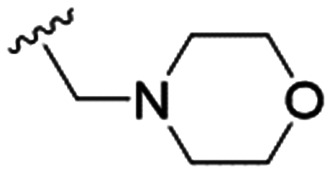	*n*-Pr	26.3 ± 3.2	>200	>7.6
31	OH	Allyl	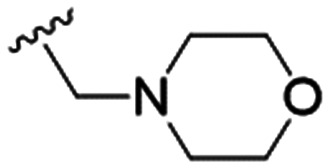	NA	51.4 ± 4.7	ND
32	OH	Allyl	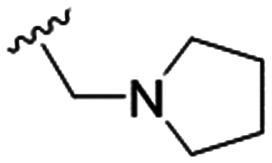	NA	43.3 ± 5.3	ND
Milt.	—			6.5 ± 3.0	119.7 ± 4.2	18.4

The substitution of the allyl sidechains with polar morpholine or piperidine groups led to increased activity in all cases 24–30, although a pyrrolidine substitution (23) did not. Substitution at position S2-B tends to result in higher antileishmanial activity (IC_50_ ranging from 3.7 to 9.7 μM) than S2-A (IC_50_ ranging from 13.2 μM to inactive). An attempt to amplify the activity increase with a double substitution (27) did not have the desired effect, instead resulting in an IC_50_ value comparable to the less potent S2-A substitutions. Unlike position S1 there does not appear to be a consistent anti-parasitic IC_50_ trend corresponding to the different p*K*_a_ values for the morpholine (least basic), piperidine and pyrrolidine (most basic) derivatives at position S2. The compounds broadly fit the trend of higher p*K*_a_ corresponding to higher mammalian cytotoxicity with pyrrolidine containing compound 23 having the worst CC_50_ value for this series.

Our next step was to investigate *ortho*-methoxy groups at position **S3** (Table 3). Deletion of the methoxy group at S3-B in compounds 33 and 34 was well-tolerated, with no effect on antileishmanial activity. The same is not true for S3-A: 35 was inactive against *L.* (*L.*) *infantum* upon deletion of this methoxy group compared to the corresponding highly active compound 25. Therefore, the methoxy group at S3-B is expendable, which could offer synthetic benefits, while that at S3-A is crucial for maintaining anti-parasitic activity.

**Table 3:** Anti-*Leishmania* (*L.*) *infantum* activity and mammalian cytotoxicity of the dehydrodieugenol B analogues, modification at S3

Comp	S1	S2A	S2B	S3A	S3B	IC_50_ (μM) ± SD	CC_50_ (μM) ± SD	SI
33	OPMB	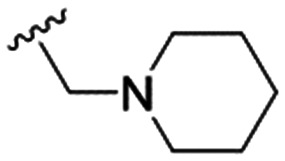	*n*-Pr	OMe	H	3.0 ± 1.1	22.7 ± 0.9	7.6
34	OPMB	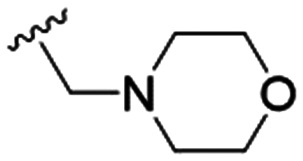	*n*-Pr	OMe	H	15.3 ± 1.9	>200	>13.1
35	OPMB	*n*-Pr	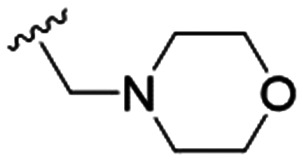	H	OMe	NA	33.8 ± 4.9	ND
36	OPMB	Allyl	Allyl	OMe	H	NA	>200	ND
37	OH	Allyl	Allyl	OMe	H	NA	123.4 ± 9.4	ND
38	OPMB	Allyl	Allyl	H	OMe	NA	>200	ND
39	OH	Allyl	Allyl	H	OMe	NA	128.6 ± 5.2	ND
Milt.	—					6.5 ± 3.0	119.7 ± 4.2	18.4

These results demonstrate that the presence of a polar functionality such as morpholine, pyrrolidine or piperidine is generally required for anti-*Leishmania* (*L.*) *infantum* activity, with 12 of the 14 active analogues containing one of these functional groups. 9 and 11 are the only exceptions but display a fairly low activity of 24.2 and 32.7 μM respectively. This polar group requirement could be linked to compound solubility and ability to permeate cell membranes; due to the nature of *Leishmania* (*L.*) *infantum* amastigotes residing in intracellular vacuoles the compound must pass through multiple cell membranes to act directly on the parasite. The presence of a free phenol at position S1 (as in the natural product itself) led to no anti-parasitic activity in every example tested, even when paired with a polar functionality as in 31 and 32, indicating the importance of a substituent in the S1 position. The series 28 to 30 attempted to investigate the effect of decreasing electron density at the benzylic S1 substituent. Electron withdrawing *para*-trifluoromethylbenzyl group showed a slightly lower IC_50_ value than strongly electron donating trimethoxybenzyl, with *para*-methoxybenzyl having an IC_50_ in between the two. However, these changes were of a small magnitude indicating electron density at this position is unlikely to play a major role in compound activity. Interestingly, the introduction of a piperidine group leads to lower IC_50_ values than morpholine substitution: compounds 26 and 33 have potent IC_50_ values of approximately 3 μM compared with the corresponding morpholine analogues 25 and 34 (7.7 and 15.3 μM respectively). However, this trend is also manifested in the mammalian cytotoxicity, with higher CC_50_ values for the morpholine analogues, which overall gives the morpholine analogues a better selectivity index.

Compound 24 emerged as the most favourable with the best selectivity index due to its high anti-parasitic activity (9.7 μM) coupled with no mammalian cytotoxicity (>200 μM). It was therefore selected for an *in silico* study of pharmacokinetic properties alongside closely related analogues 28 and 34. An analysis of pharmacokinetic properties and structural alerts for compounds 24, 28 and 34 was conducted using ADMETlab 2.0 (Table 4).^13^ The predictions were performed to explore the safety and drug-likeness profile of these analogues. The physicochemical property analysis of 24 predicts a log *P* value of 5.18, a close to optimum log *D* value of 3.82 and water solubility (log *S*) of −5.27, which falls into the moderate solubility class – an important characteristic that relates to oral bioavailability which we show here to be high (≥30%). Polarity of 24 is also predicted to be favourable (0 < TPSA < 140 Å^2^). All these characteristics are improved in 24 compared to the related analogues 28 and 34, further justification for choosing 24 as the best compound from this series (see the ESI† for further ADMET analysis on 24).

**Table 4:** *In silico* predictions of physicochemical, structural and ADMET parameters

Properties	24	28	34
Molecular weight	505.25	507.26	477.25
Log *P*	5.18	5.65	5.69
Log *D*	3.82	4.12	4.17
Log *S*	−5.27	−6.03	−6.07
TPSA	58.62	58.62	49.39
Caco-2 permeability	−5.03, optimal	−5.46, moderate	−5.30, moderate
Pgp inhibitor	Yes	Yes	Yes
Pgp substrate	No	No	No
HIA (≥30%)	Yes	Yes	Yes
Oral bioavailability	≥30% F	≥30% F	≥30% F
BBB penetration	No	No	No
Distribution (*V*_D_) (L K^−1^)	0.72, optimal	0.72, optimal	0.95, optimal
CYP1A2 inhibitor	No	No	No
CYP2C19 inhibitor	Yes	Yes	Yes
CYP2C9 inhibitor	Yes	No	Yes
CYP2D6 inhibitor	No	No	No
CYP3A4 inhibitor	Yes	Yes	Yes
Clearance (mg min^−1^ kg^−1^)	11.6, moderate	11.6, moderate	11.7 moderate
hERG blockers	Yes	Yes	Yes
Human hepatotoxicity	No	No	No
AMES toxicity	No	No	No
DILI	Moderate	Moderate	Moderate
Lipinski rules	Fail	Fail	Pass
PAINs	0 alerts	0 alerts	0 alerts

The series is predicted to have a high Human Intestinal Absorption (HIA) being ≥30%, moderate (compounds 28, 34) to optimum (compound 24) Caco-2 permeability – another model of intestinal absorption, and pleasingly no permeability to the blood–brain barrier, which could lead to side-effects in the nervous system.^14^ The cytochrome P450 superfamily of enzymes are responsible for compound metabolism; inhibition of these enzymes can induce adverse effects and toxicity due to accumulation of drug metabolites.^15^ Of the five main isoforms (CYP1A2, CYP2C19, CYP2C9, CYP2D6, CYP3A4), 24 is proposed to be an inhibitor of three with CYP2C9 and CYP3A4 having only a weak positive result.

Compound 24 exceeds the suggested limit of flexibility (number of rotatable bonds < 11) having 12 rotatable bonds, and lies close to the recommended boundary for size criteria (100 < MW < 600) and lipophilicity (0 < log *P* < 3.0), although values up to 5.0 are still considered reasonable by Lipinski's rules.^16^ Compounds 24 and 28 fail Lipinski's rule due to the high molecular weight (>500), although they are close to the boundary at 505 and 507 respectively. This may imply flexibility and size issues which should be addressed with future structural optimization. Otherwise, compound 24 demonstrated favourable predictions for toxicity, with no indication of human hepatotoxicity or AMES mutagenicity, and only moderate drug induced liver injury measurements. It is also important to note there were no structural similarities to pan-assay interference compounds (PAINs).

According to Drugs for Neglected Diseases *initiative* (DND*i*), a new hit compound for Visceral Leishmaniasis, should: i) be synthesized in no more than 8 steps; ii) present IC_50_ lower than 10 μM in amastigotes; iii) have selectivity index higher than 10; iv) exhibit an appropriate *in silico* ADME profile and, v) show no structural alerts.^11,17^ Based on these criteria and the results obtained herein, compound 24 can be considered as a promising hit for Visceral Leishmaniasis.

## Experimental

### General experimental procedures

MTT was purchased from Molecular Probes® (Invitrogen™). Fetal bovine serum (FBS) was obtained from Gibco. All absorbance readings were performed using the FilterMax F5 Multi-Mode Microplate Reader spectrofluorimeter (Molecular Devices). Proton (^1^H) NMR spectra were recorded at 400 or 500 MHz and carbon (^13^C) NMR spectra at 101 or 126 MHz with ^1^H decoupling. Spectra were recorded on Bruker AVIIIHD 400 or Bruker AVIIIHD 500 spectrometers with CDCl_3_ as reference. High-resolution mass spectra (HRMS) were recorded by the Departmental Mass Spectrometry Service, University of Oxford on a Thermo Scientific Exactive Mass Spectrometer (using a Waters Equity autosampler and pump) for electrospray ionisation (ESI).

### Animals

Golden hamsters (*Mesocricetus auratus*) and BALB/c mice were obtained from the animal breeding facility of the Instituto Adolfo Lutz – SP and maintained in sterilized cages with controlled environment. All animal procedures were performed in accordance with the Guidelines for Care and Use of Laboratory Animals of the Brazilian National Council of Animal Experimentation (COBEA) and had the approval of the Animal Ethics Committee (CEUA IMT-USP 000404A) from University of São Paulo and Instituto Adolfo Lutz – Secretary of Health of Sao Paulo State (CEUA 05/2018).

### Parasite and mammalian cell maintenance

*Leishmania* (*L.*) *infantum* (MHOM/BR/1972/LD) amastigotes were obtained by differential centrifugation from golden hamsters (*Mesocricetus auratus*) spleens with 60–70 days of infection and the number of parasites was determined by the Stauber method.^18^ Murine conjunctive fibroblasts NCTC clone 929 (ATCC) were maintained in RPMI-1640 medium supplemented with 10% FBS at pH 7.2 and 37 °C in a humidified incubator with 5% CO_2_. Peritoneal macrophages were by obtained by washing the peritoneal cavity of BALB/c mice with RPMI-1640 medium supplemented with 10% FBS, pH 7.2 and maintained at 37 °C in a humidified incubator with 5% CO_2_.

### Anti-*L.* (*L.*) *infantum* activity *in vitro*

Peritoneal macrophages (1 × 10^5^/well) were added in 16-well slide chambers (NUNC) and incubated overnight at 37 °C with 5% CO_2_. Subsequently, the cells were infected with *L.* (*L.*) *infantum* amastigotes (10 : 1 amastigotes/macrophage) for 24 h, treated with compounds serially diluted (6.25 to 50 μM) and incubated for 96 h at 37 °C with 5% CO_2_. Miltefosine was used as standard drug and untreated cells as a negative control. Slides were fixed with methanol, stained with Giemsa and observed under a light microscope.^19^ The 50% inhibitory concentration (IC_50_) was determined by the infection index (number of infected macrophages × amastigotes/total macrophages).^12^

### Mammalian toxicity *in vitro*

NCTC cells (6 × 10^4^/well) were added to 96-well plates and maintained at 37 °C in a 5% CO_2_ humidified incubator for 96 h with compounds serially diluted (1.6 to 200 μM). The 50% cytotoxic concentration (CC_50_) was determined using the MTT colorimetric method and the selectivity index was calculated by the ratio: CC_50_ against NCTC cells/IC_50_ against amastigotes.^20^

### *In silico* analysis

Predictions were performed using the web server ADMETlab 2.0. This platform includes several models for physicochemical properties, solubility, pharmacokinetic/pharmacodynamic parameters, drug-likeness profile and structural alerts for pan-assay interference compounds (PAINS).^13^

### Statistical analysis

The determination of CC_50_ and IC_50_ values was performed by sigmoid dose–response curves using the Graph Pad Prism 5 software. The samples were tested in duplicate and the assays were reproduced at least twice.

## Conclusions

Among 39 tested analogues, 14 were active against the intracellular amastigote form of *Leishmania* (*L.*) *infantum*. SAR analysis suggests that polar functionalities are crucial for the activity of this family, with morpholine substituted analogues displaying the best SI. Derivative 24 was the most promising compound from this series, with excellent potency and selectivity, and can be considered a promising hit for VL. It is synthesized in 5 steps in the longest linear sequence (and 6 steps in total), displays promising bioactivity (9.7 μM), low mammalian toxicity (SI > 20.6), and meets the predicted ADME criteria.

## Author contributions

Conceptualization, AGT, EAA and MMH; methodology, MA, HA, CES, SS, PP; software, MA, HA; investigation, MA, HA, CES, SS, PP; resources, EEA and AGT; data curation, EAA and AGT; writing—original draft preparation, MA, HA, writing—review and editing, AGT, EAA, HA and MMH. All authors have read and agreed to the published version of the manuscript.

## Conflicts of interest

There are no conflicts to declare.

## Supplementary Material

MD-014-D3MD00081H-s001
