# Supplementary material for: Biological activity and structure–activity relationship of dehydrodieugenol B analogues against visceral leishmaniasis
Source: RSC Med Chem. 2023 May 26;14(7):1344–50. doi: 10.1039/d3md00081h (PMC10357944; doi:10.1039/d3md00081h)
Supplement: MD-014-D3MD00081H-s001 [file MD-014-D3MD00081H-s001.pdf]

Electronic Supplementary Material (ESI) for RSC Medicinal Chemistry.
This journal is © The Royal Society of Chemistry 2023

SUPPORTING INFORMATION

Biological Activity and Structure−Activity Relationship of Dehydrodieugenol B Analogues against Visceral Leishmaniasis

Maiara Amaral,[1,2‡] Hannah Asiki,[3‡] Claire E. Sear,[3] Snigdha Singh,[3] Pauline Pieper,[3] Marius M. Haugland,[3] Edward A. Anderson[3*] and Andre G. Tempone[2*]

[1] Instituto de Medicina Tropical, Faculdade de Medicina, Universidade de Sao Paulo, Sao Paulo - 05403-000, Brazil.

[2] Centre for Parasitology and Mycology, Instituto Adolfo Lutz, São Paulo -01246-000, Brazil.

[3] Chemistry Research Laboratory, Department of Chemistry, University of Oxford, 12 Mansfield Road, Oxford, OX1 3TA, U.K.

‡These authors contributed equally.

*Corresponding authors:

AGT (andre.tempone@ial.sp.gov.br);

EAA (edward.anderson@chem.ox.ac.uk).

Contents

1. *In silico* ADMET / Physicochemical Analysis 2
2. General Experimental Considerations 2
3. General Synthetic Methods 3
4. Synthesis and characterization of compounds 4
5. Copies of ^{1}H NMR spectra 22
6. References 32

1. *In silico* ADMET / Physicochemical Analysis

Analysis of the ADMET and physicochemical properties of compound **24** was carried out using the ADMETlab 2.0 programme[1]. The output includes a bioavailability radar representing ideal drug-likeness (Figure S1).

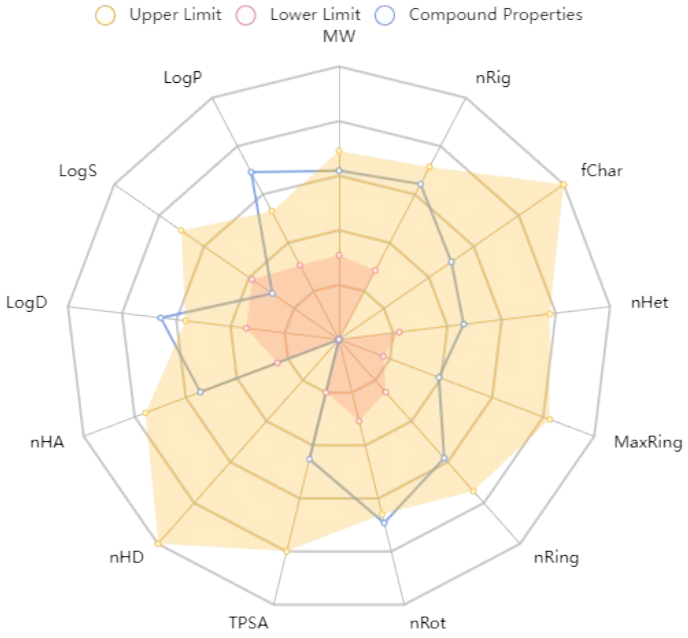

Figure S1 - Compound **24** bioavailability radar, obtained using the ADMETlab 2.0 programme. The light orange area represents the ideal drug-likeness values for each property, labelled here clockwise from the top: Size (MW 100 to 600 g/mol), Number of rigid bonds (0 to 30), Formal charge (-4 to +4), Number of heteroatoms (1 to 15), Number of atoms in the biggest ring (0 to 18), Number of rings (0 to 6), Number of rotatable bonds (0 to 11), Topological Polar Surface Area (0 to 140), Number of H bond donors (0 to 7), Number of H bond acceptors (0 to 12), LogD (1 to 3), LogS (-4 to 0.5), LogP (0 to 3).

2. General Experimental Considerations

Materials/procedures: Dichloromethane (CH_2Cl_2), dioxane, tetrahydrofuran (THF), and *N*,*N*-dimethylformamide (DMF) were dried by passing through an activated alumina column under argon in an MBraun SPS-800 solvent dispenser. All other reagents were used as received from the suppliers. Caesium carbonate and copper(I) chloride were weighed out in a glove box under nitrogen. Petrol refers to the fraction of petroleum ether which boils in the range 40-60 °C. Brine refers to a saturated aqueous solution of NaCl. All air- or moisture-sensitive reactions were carried out with anhydrous solvents in glassware dried under vacuum and heated with a heat gun under an inert atmosphere of nitrogen. For reactions that require heating, an oil bath was employed. The temperature was monitored *via* a temperature probe plugged into the stirrer plate.

Chromatography: Thin-layer chromatography was performed on pre-coated aluminium-backed plates (Merck Kieselgel 60 F_{254} plates), which were visualised with UV fluorescence (254 nm) and/or staining with potassium(VII) manganate, vanillin, phosphomolybdic acid or ninhydrin followed by heating with a heat gun. Column chromatography refers to normal phase column chromatography unless specified otherwise and was performed manually using Geduran® Silicagel 60 (40-63 µm), under a positive

pressure of nitrogen with the solvent system used in parentheses. Retention factors (R_f) are reported with the solvent system in parentheses.

Infrared Spectroscopy: Infrared spectra were recorder on a Bruker Tensor 27 Fourier transform spectrometer, as a thin film on a diamond ATR module. Wavelengths of maximum absorbance (ν_{max}) are quoted in cm^{-1}. Only selected, characteristic IR absorption data are provided for each compound.

NMR Spectroscopy: Proton (^1H) NMR spectra were recorded at 400 or 500 MHz and carbon (^{13}C) NMR spectra at 101 or 126 MHz with ^1H decoupling. Spectra were recorded on Bruker AVIIIHD 400 or Bruker AVIIIHD 500 spectrometers. Chemical shifts (δ_H and δ_C) are expressed in parts per million (ppm), referenced to the residual solvent peak of $CDCl_3$ (7.26 ppm). Coupling constants (J) are reported to the nearest 0.1 Hz. Splitting patterns are described using the following abbreviations: s (singlet), d (doublet), dd (doublet of doublets), ddt (doublet of doublet of triplets), dt (doublet of triplets), t (triplet), q (quartet), m (multiplet) and br (broad).

Mass Spectrometry: Low-resolution mass spectra were recorded on a Micromass LCT Premier Open Access using electrospray ionisation (ESI). High-resolution mass spectra (HRMS) were recorded by the Departmental Mass Spectrometry Service, University of Oxford on a Thermo Scientific Exactive Mass Spectrometer (using a Waters Equity autosampler and pump) for electrospray ionisation (ESI). High resolution values are calculated to 4 decimal places from the molecular formula, and all values are within a tolerance of 5 ppm.

3. General Synthetic Methods

General Method A: MOM protection of phenols

A solution of phenol (1.0 equiv.) in CH_2Cl_2 (0.2 M) was cooled to 0 °C and chloromethyl methyl ether was added (1.5 equiv.), followed by dropwise addition of DIPEA (2.0 equiv.) The resulting solution was allowed to warm to room temperature and stirred until the reaction was complete. The reaction mixture was washed successively with aqueous 0.5 M HCl, H_2O, aqueous 1 M NaOH and brine, dried over $MgSO_4$, and evaporated to dryness.

General Method B: *Ortho*-Iodination of protected phenols

To a solution of protected phenol (1.0 equiv.) in anhydrous THF (0.6 M) was added tetramethylethylenediamine (1.5 equiv.) The solution was cooled to −78 °C then *sec*-butyllithium solution (1.4 M in hexane, 1.5 equiv.) was added dropwise and stirred for 3 h. Iodine (1.5 equiv.) was added slowly as a solution in anhydrous THF (5 mL), and after 10 mins was allowed to warm to room temperature and stirred until the reaction was complete. The resulting solution was quenched with $Na_2S_2O_3$ (aq., sat.), diluted with H_2O, CH_2Cl_2 and the layers separated. The aqueous was extracted with CH_2Cl_2, the combined organic extracts washed with NH_4Cl (aq., sat.), brine, dried over $MgSO_4$ and evaporated to dryness.

General Method C: MOM deprotection of phenols

To a solution of protected phenol (1.0 equiv.) in MeOH (0.03 M) was added aqueous 2 M HCl (0.1 *v/v*) and left stirring until the reaction was complete. The reaction mixture was basified to pH 5 with aqueous 1 M NaOH, diluted with EtOAc and the layers separated. The aqueous was extracted with EtOAc, combined organic extracts washed with brine, dried over $MgSO_4$ and evaporated to dryness.

General Method D: Benzylation of phenols

To a solution of phenol (1.0 equiv.) and potassium carbonate (2.5 equiv.) in DMF (0.13 M) was added benzyl chloride (1.2 equiv.) and heated to 80 °C while stirring until the reaction was complete. The reaction mixture was diluted with EtOAc, H_2O and separated. The organic extract was washed with H_2O, brine, dried over $MgSO_4$ and evaporated to dryness.

General Method E(a,b): Ullmann cross-coupling

A vial containing halide (1.0 equiv.), CuCl (0.5 equiv.) and caesium carbonate (2.0 equiv.), was capped with a rubber septum then evacuated and backfilled with N_2 (× 3). To this was added phenol (2 equiv.), *N*-methyl-2-pyrrolidone (5 M *wrt* halide) and 2,2,6,6-tetramethyl-3,5-heptanedione (0.5 equiv.) *via* syringe, then evacuated and backfilled with N_2 (× 3) before addition of de-gassed 1,4-dioxane (2.5 M *wrt* halide) (a only). The vial was capped and taped, and the reaction mixture heated to 80 °C (a) or 120 °C (b) until complete. The solution was diluted with Et_2O, washed with 35% ammonia solution, brine, dried over Na_2SO_4 and evaporated to dryness.

General Method F: PMB deprotection of phenols

To a solution of PMB-protected phenol (1 equiv.) in EtOH (0.05 M) was added aqueous 1 M HCl (0.02 M) and heated to 80 °C while stirring until the reaction was complete. The reaction mixture was basified to pH 7 with aqueous 1 M NaOH, extracted with EtOAc, combined organic extracts washed with brine, dried over Na_2SO_4 and evaporated to dryness.

General Method G: Acylation of phenols

To a solution of phenol (1.0 equiv.) in pyridine (23.0 equiv.) was added acetic anhydride (7.5 equiv.) and heated to 100 °C while stirring for 2 h. The reaction mixture was cooled to room temperature and quenched with ice. The solution was acidified to pH 7 by dropwise addition of aqueous 1 M HCl, extracted with EtOAc, combined organic extracts washed with aqueous 1 M HCl, brine, dried over $MgSO_4$ and evaporated to dryness.

General Method H: Reductive amination

To a solution of aldehyde (1.0 equiv.) and amine (1.0 equiv.) in anhydrous THF (0.13 M *wrt* aldehyde) was added $NaBH(OAc)_3$ (1.5 equiv.) and left stirring until the reaction was complete. The reaction mixture was quenched with $NaHCO_3$ (sat. aq.), extracted with Et_2O, combined organic extracts washed with brine, dried over Na_2SO_4 and evaporated to dryness.

4. Synthesis and characterization of compounds

Final compounds **1** – **3**, **8** – **10**, **13**, **14**, **17** – **22**, and **36** – **39** were prepared as previously published[2].

Synthesis of the remaining compounds are presented below.

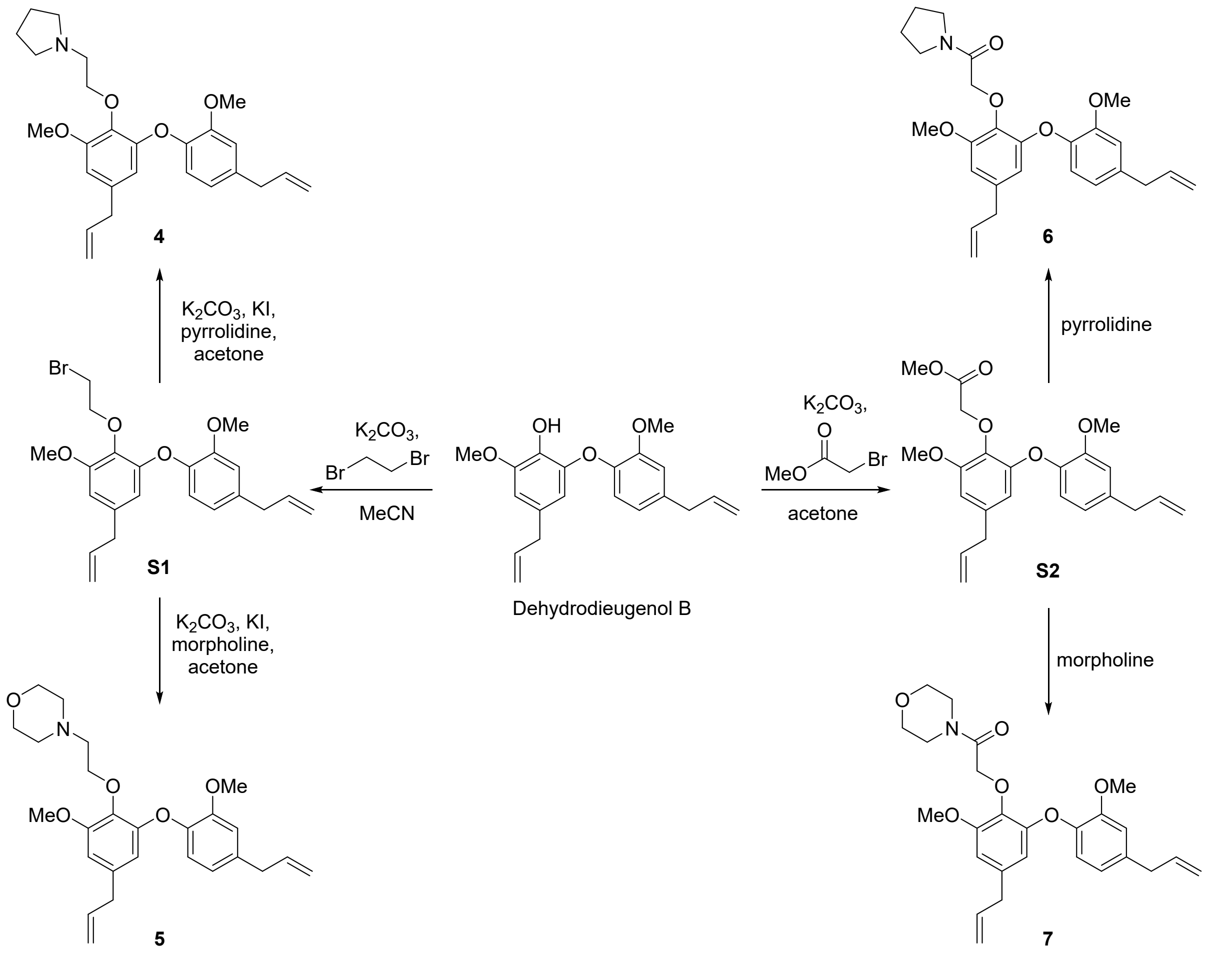

Scheme 1: *Synthetic routes to analogues **4 – 7**. Dehydrodieugenol B prepared as previously published*[2].

5-Allyl-1-(4-allyl-2-methoxyphenoxy)-2-(2-bromoethoxy)-3-methoxybenzene, S1

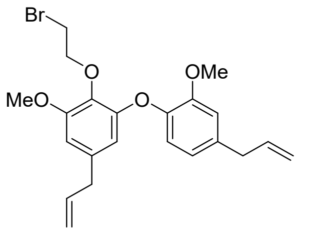

To a solution of dehydrodieugenol B, (109 mg, 0.330 mmol, 1.0 equiv.) and potassium carbonate (138 mg, 1.00 mmol, 3.0 equiv.) in acetonitrile (1.70 mL) was added 1,2-dibromoethane (140 µL, 1.65 mmol, 5.0 equiv.) and heated to 80 °C while stirring until the reaction was complete (18 h). The solution was cooled to room temperature, filtered, and evaporated to dryness. Purification *via* column chromatography (1 : 0 → 4 : 1 pentane / EtOAc) afforded the title compound (72 mg, 0.17 mmol, 50%) as a pale yellow oil; R_f 0.40 (4 : 1 pentane / EtOAc); IR (thin film, $ν_{max}$ / cm^{-1}) 2935, 2360, 1638, 1587, 1595, 1424, 1266, 1187, 1152, 1092; ^{1}H NMR (400 MHz, $CDCl_3$) δ 6.74 (d, *J* = 8.1 Hz, 1H), 6.73 (d, *J* = 1.9 Hz, 1H), 6.63 (dd, *J* = 8.1, 1.9 Hz, 1H), 6.40 (d, *J* = 1.9 Hz, 1H), 6.19 (d, *J* = 1.9 Hz, 1H), 5.91 (ddt, *J* = 16.9, 10.2, 6.7 Hz, 1H), 5.86 – 5.74 (m, 1H), 5.07 – 4.92 (m, 4H), 4.27 – 4.17 (m, 2H), 3.78 (s, 3H), 3.75 (s, 3H), 3.51 – 3.43 (m, 2H), 3.30 (dt, *J* = 6.7, 1.6 Hz, 2H), 3.16 (dt, *J* = 6.7, 1.5 Hz, 2H); ^{13}C NMR (101 MHz, $CDCl_3$) δ 153.5, 150.7, 150.6, 143.7, 137.4, 137.0, 136.3, 136.2, 136.1, 120.8, 119.7, 116.0, 115.9,

113.0, 111.0, 107.2, 72.7, 56.1, 55.9, 40.1, 40.0, 29.5; HRMS (ESI+) calc. for $C_{22}H_{25}O_4Br$ $[M+H]^+$ 433.1009, found 433.1010.

1-(2-(4-Allyl-2-(4-allyl-2-methoxyphenoxy)-6-methoxyphenoxy)ethyl)pyrrolidine, 4

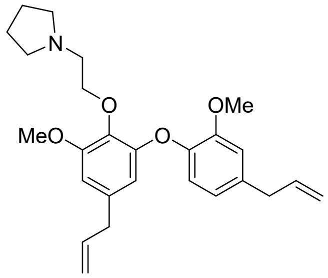

To a solution of 5-allyl-1-(4-allyl-2-methoxyphenoxy)-2-(2-bromoethoxy)-3-methoxybenzene, **S1** (35 mg, 0.080 mmol, 1.0 equiv.) in acetone (5.0 mL) was added potassium carbonate (112 mg, 0.800 mmol, 10 equiv.), potassium iodide (27 mg, 0.16 mmol, 2.0 equiv.), pyrrolidine (34 µL, 0.40 mmol, 5.0 equiv.) and heated to 75 °C while stirring until the reaction was complete (18 h). The solution was cooled to room temperature and diluted with EtOAc (10 mL), washed with aqueous 0.1 M HCl, brine, dried over $MgSO_4$ and evaporated to dryness. Purification *via* column chromatography (1 : 0 → 9 : 1 CH_2Cl_2 / MeOH) afforded the title compound (8 mg, 0.02 mmol, 23%) as a pale yellow oil; R_f 0.23 (19 : 1 CH_2Cl_2 / MeOH); IR (thin film, ν_{max} / cm^{-1}) 2919, 2850, 2361, 1589, 1507, 1463, 1428, 1266, 1212, 1154; ^{1}H NMR (400 MHz, $CDCl_3$) δ 6.77 – 6.82 (m, 2H), 6.70 (dd, *J* = 8.1, 2.0 Hz, 1H), 6.47 (d, *J* = 2.0 Hz, 1H), 6.26 (d, *J* = 1.9 Hz, 1H), 6.03 – 5.82 (m, 2H), 5.13 – 4.98 (m, 4H), 4.29 (t, *J* = 5.5 Hz, 2H), 3.85 (s, 3H), 3.82 (s, 3H), 3.37 (dt, *J* = 6.7, 1.6 Hz, 2H), 3.23 (dt, *J* = 6.6, 1.6 Hz, 2H), 3.14 – 3.05 (m, 2H), 3.04 – 2.89 (m, 4H), 2.03 – 1.73 (m, 4H); ^{13}C NMR (101 MHz, $CDCl_3$) δ 153.8, 150.6, 150.5, 144.4, 137.6, 137.2, 137.1, 136.0, 135.8, 120.9, 119.3, 116.1, 116.0, 113.1, 111.7, 107.6, 71.1, 56.2, 56.1, 55.4, 54.3, 40.2, 40.1; HRMS (ESI+) calc. for $C_{26}H_{33}O_4N$ $[M+H]^+$ 424.2482, found 424.2479.

4-(2-(4-Allyl-2-(4-allyl-2-methoxyphenoxy)-6-methoxyphenoxy)ethyl)morpholine, 5

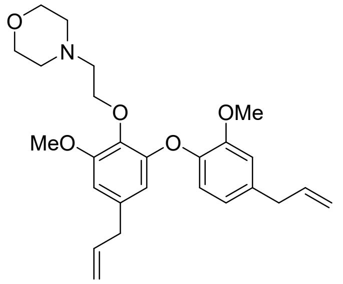

To a solution of 5-allyl-1-(4-allyl-2-methoxyphenoxy)-2-(2-bromoethoxy)-3-methoxybenzene, **S1** (35 mg, 0.080 mmol, 1.0 equiv.) in acetone (5.0 mL) was added potassium carbonate (112 mg, 0.800 mmol, 10 equiv.), potassium iodide (27 mg, 0.16 mmol, 2.0 equiv.), morpholine (35 µL, 0.40 mmol, 5.0 equiv.) and heated to 75 °C while stirring until the reaction was complete (18 h). The solution was cooled to room temperature and diluted with EtOAc (10 mL), washed with aqueous 0.1 M HCl, brine, dried over $MgSO_4$ and evaporated to dryness. Purification *via* column chromatography (1 : 0 → 0 : 1 pentane / EtOAc) afforded the title compound (30 mg, 0.070 mmol, 83%) as a colourless oil; R_f 0.30 (3 : 7 pentane / EtOAc); IR (thin film, ν_{max} / cm^{-1}) 2921, 2851, 1586, 1505, 1452, 1425, 1330, 1265, 1151, 1092; ^{1}H NMR (400 MHz, $CDCl_3$) δ 6.78 (d, *J* = 2.0 Hz, 1H), 6.75 (d, *J* = 8.1 Hz, 1H), 6.67 (dd, *J* = 8.1, 1.9 Hz, 1H), 6.48 (d, *J* = 1.9 Hz, 1H), 6.31 (d, *J* = 1.9 Hz, 1H), 6.04 – 5.81 (m, 2H), 5.14 – 4.98 (m,

4H), 4.12 (t, J = 5.7 Hz, 2H), 3.84 (s, 3H), 3.83 (s, 3H) 3.69 – 3.62 (m, 4H), 3.36 (dd, J = 6.7, 1.6 Hz, 2H), 3.24 (dt, J = 6.6, 1.5 Hz, 2H), 2.69 (t, J = 5.7 Hz, 2H), 2.53 – 2.46 (m, 4H); ^{13}C NMR (101 MHz, $CDCl_3$) δ 153.8, 150.5, 150.3, 144.5, 137.5, 137.4, 137.2, 135.8, 135.8, 120.8, 118.9, 116.1, 116.0, 113.0, 112.0, 107.7, 70.5, 67.1, 58.4, 56.1, 56.0, 53.9, 40.2, 40.1; HRMS (ESI+) calc. for $C_{26}H_{33}O_5N$ $[M+H]^+$ 440.2431, found 440.2422.

Methyl 2-(4-allyl-2-(4-allyl-2-methoxyphenoxy)-6-methoxyphenoxy)acetate, S2

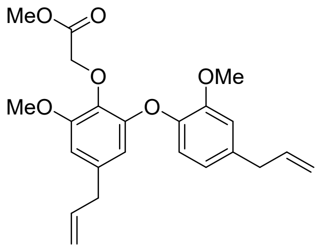

To a solution of dehydrodieugenol B, (50 mg, 0.15 mmol, 1.0 equiv.) and potassium carbonate (32 mg, 0.23 mmol, 1.5 equiv.) in acetone (0.25 mL) was added methyl bromoacetate (22 µL, 0.23 mmol, 1.5 equiv.) and left stirring for 20 h. An additional portion of potassium carbonate (32 mg, 0.23 mmol, 1.5 equiv.) and methyl bromoacetate (22 µL, 0.23 mmol, 1.5 equiv.) were added and stirred until the reaction was complete (4 h). The solution was filtered through a pad of celite, dried over $MgSO_4$ and evaporated to dryness. Purification *via* column chromatography (1 : 0 → 4 : 1 pentane / EtOAc) afforded the title compound (60 mg, 0.15 mmol, 98%) as a colourless oil; R_f 0.31 (4 : 1 pentane / EtOAc); IR (thin film, ν_{max} / cm^{-1}) 2919, 2850, 2361, 1766, 1738, 1589, 1507, 1428, 1212, 1152; ^{1}H NMR (400 MHz, $CDCl_3$) δ 6.84 (d, J = 8.0 Hz, 1H), 6.78 (d, J = 1.9 Hz, 1H), 6.70 (dd, J = 8.0, 2.0, 1H), 6.46 (d, J = 1.9 Hz, 1H), 6.23 (d, J = 1.8 H, 1H), 5.97 (ddt, J = 16.9, 10.1, 6.7 Hz, 1H), 5.86 (ddt, J = 17.5, 9.5, 6.6 Hz, 1H), 5.17 – 4.95 (m, 4H), 4.68 (s, 2H), 3.84 (s, 3H), 3.80 (s, 3H), 3.73 (s, 3H), 3.36 (dt, J = 6.6, 1.5 Hz, 2H), 3.22 (dt, J = 6.7, 1.5, 2H); ^{13}C NMR (101 MHz, $CDCl_3$) δ 170.0, 153.2, 150.8, 150.5, 143.7, 137.5, 137.1, 136.5, 136.1, 136.0, 120.9, 120.1, 116.1, 116.0, 113.2, 111.0, 107.3, 69.8, 56.3, 56.0, 52.0, 40.2, 40.1; HRMS (ESI+) calc. for $C_{23}H_{26}O_6$ $[M+H]^+$ 399.1802, found 399.1795.

2-(4-Allyl-2-(4-allyl-2-methoxyphenoxy)-6-methoxyphenoxy)-1-(pyrrolidin-1-yl)ethan-1-one, 6

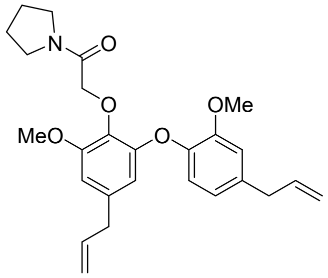

A solution of methyl 2-(4-allyl-2-(4-allyl-2-methoxyphenoxy)-6-methoxyphenoxy)acetate, **S2** (40 mg, 0.10 mmol, 1.0 equiv.) in pyrrolidine (44 µL, 0.52 mmol, 5.2 equiv.) was heated to 75 °C while stirring until the reaction was complete (24 h). The solution was cooled to room temperature and diluted with EtOAc (5 mL), washed with NH_4Cl (sat., aq.), dried over $MgSO_4$ and evaporated to dryness. Purification *via* column chromatography (1 : 0 → 3 : 7 pentane / EtOAc) afforded the title compound (41 mg, 0.092 mmol, 92%) as a pale yellow oil; R_f 0.14 (1 : 1 pentane / EtOAc); IR (thin film, ν_{max} / cm^{-1}) 2923, 1639, 1588, 1505, 1451, 1426, 1265, 1212, 1152, 1091; ^{1}H NMR (400 MHz, $CDCl_3$) δ 6.83 – 6.75 (m, 2H), 6.69 (dd, J = 8.1, 1.9 Hz, 1H), 6.47 (d, J = 1.9 Hz, 1H), 6.25 (d, J = 1.7 Hz, 1H), 6.04 – 5.79 (m, 2H), 5.15 – 4.97

(m, 4H), 4.63 (s, 2H), 3.85 (s, 3H), 3.81 (s, 3H), 3.59 (t, *J* = 6.5 Hz, 2H), 3.44 (t, *J* = 6.6 Hz, 2H), 3.36 (dd, *J* = 6.7, 1.5 Hz, 2H), 3.22 (dd, *J* = 6.7, 1.5 Hz, 2H), 1.80 (dt, *J* = 28.5, 5.8 Hz, 4H); ^{13}C NMR (101 MHz, $CDCl_3$) δ 167.1, 153.6, 150.6, 150.5, 144.2, 137.5, 137.1, 136.4, 136.3, 136.2, 121.0, 119.5, 116.1, 116.0, 113.1, 111.5, 107.6, 73.0, 56.3, 56.1, 46.2, 46.2, 40.2, 40.1, 26.4, 24.0; HRMS (ESI+) calc. for $C_{26}H_{31}O_5N$ $[M+H]^+$ 438.2275, found 438.2271.

2-(4-Allyl-2-(4-allyl-2-methoxyphenoxy)-6-methoxyphenoxy)-1-morpholinoethan-1-one, 7

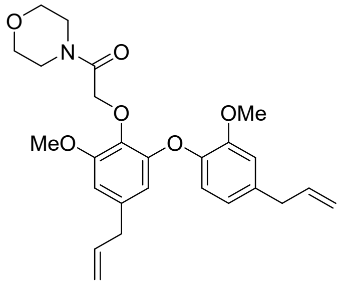

A solution of methyl 2-(4-allyl-2-(4-allyl-2-methoxyphenoxy)-6-methoxyphenoxy)acetate, **S2** (40 mg, 0.10 mmol, 1.0 equiv.) in morpholine (46 µL, 0.52 mmol, 5.2 equiv.) was heated to 60 °C while stirring until the reaction was complete (24 h). The solution was cooled to room temperature and diluted with EtOAc (5 mL), washed with NH_4Cl (sat., aq.), dried over $MgSO_4$ and evaporated to dryness. Purification *via* column chromatography (1 : 0 → 3 : 7 pentane / EtOAc) afforded the title compound (46 mg, 0.10 mmol, 99%) as a colourless oil; R_f 0.29 (1 : 1 pentane / EtOAc); IR (thin film, v_{max} / cm^{-1}) 2921, 2854, 1649, 1589, 1506, 1452, 1267, 1212, 1152, 1091; ^{1}H NMR (400 MHz, $CDCl_3$) δ 6.82 – 6.75 (m, 2H), 6.70 (dd, *J* = 8.1, 1.9 Hz, 1H), 6.48 (d, *J* = 1.9 Hz, 1H), 6.25 (d, *J* = 1.9 Hz, 1H), 5.97 (ddt, *J* = 16.9, 10.2, 6.7 Hz, 1H), 5.92 – 5.80 (m, 1H), 5.14 – 4.97 (m, 4H), 4.66 (s, 2H), 3.85 (s, 3H), 3.82 (s, 3H), 3.79 – 3.72 (m, 2H), 3.65 – 3.59 (m, 2H), 3.58 – 3.51 (m, 4H), 3.37 (dt, *J* = 6.7, 1.7 Hz, 2H), 3.23 (dt, *J* = 6.7, 1.6 Hz, 2H); ^{13}C NMR (101 MHz, $CDCl_3$) δ 167.1, 154.4, 150.0, 144.4, 137.4, 137.0, 136.8, 136.5, 136.1, 121.0, 119.5, 116.2, 116.2, 113.1, 111.3, 107.5, 72.7, 67.1, 66.9. 56.2, 56.0, 46.3, 42.5, 40.2, 40.1; HRMS (ESI+) calc. for $C_{26}H_{31}O_6N$ $[M+H]^+$ 454.2224, found 454.2224.

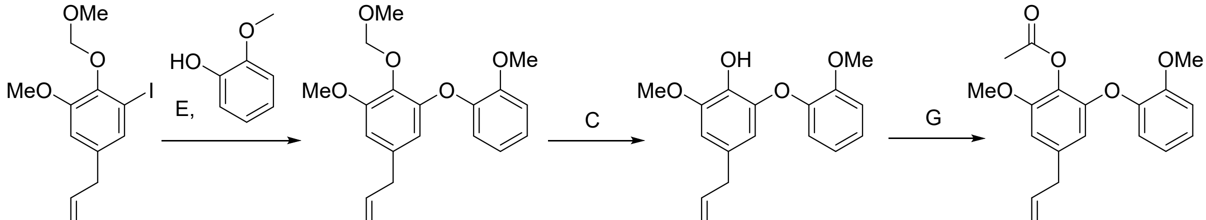

***Scheme 2:** Synthetic route to analogues **11** and **15.** 5-Allyl-1-iodo-3-methoxy-2-(methoxymethoxy)benzene prepared as previously published*[2].

5-Allyl-1-methoxy-2-(methoxymethoxy)-3-(2-methoxyphenoxy)benzene, S3

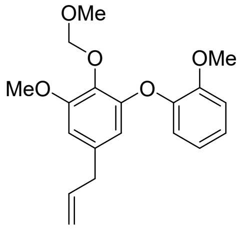

The product was prepared according to General Method E(a) from 5-allyl-1-iodo-3-methoxy-2(methoxymethoxy)benzene (81 mg, 0.24 mmol) and guaiacol (53 µL, 0.49 mmol) The reaction was complete after 72 h. Purification *via* column chromatography (1 : 0 → 4 : 1 petrol / EtOAc) afforded the title compound (22 mg, 0.067 mmol, 28%) as a colourless oil; R_f 0.22 (9 : 1 petrol / EtOAc); IR (thin film, ν_{max} / cm^{-1}) 2936, 2838, 1638, 1501, 1454, 1333, 1302, 1255, 1177, 1079; ^{1}H NMR ($CDCl_3$, 400 MHz) δ 7.12 – 7.03 (m, 1H), 6.98 (d, *J* = 8.1 Hz, 1H), 6.94 – 6.83 (m, 2H), 6.50 (d, *J* = 1.9 Hz, 1H), 6.29 (d, *J* = 1.9 Hz, 1H), 5.95 – 5.80 (m, 1H), 5.16 (s, 2H), 5.08 – 4.98 (m, 2H), 3.86 (s, 3H), 3.84 (s, 3H), 3.56 (s, 3H), 3.24 (d, *J* = 6.7 Hz, 2H); ^{13}C NMR (101 MHz, $CDCl_3$) δ 153.8, 150.8, 150.6, 145.8, 137.1, 136.3, 134.7, 124.1, 121.1, 119.5, 116.1, 112.7, 111.6, 107.5, 98.5, 57.2, 56.1, 56.0, 40.2; HRMS (ESI+) calc. for $C_{19}H_{22}O_5$ $[M+Na]^+$ 353.1359, found 353.1356.

4-Allyl-2-methoxy-6-(2-methoxyphenoxy)phenol, 15

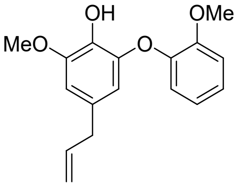

The product was prepared according to General Method C from 5-allyl-1-methoxy-2(methoxymethoxy)-3-(2-methoxyphenoxy)benzene, **S3** (22 mg, 0.070 mmol). The reaction was complete after 20 h. Purification *via* column chromatography (4 : 1 petrol / EtOAc) afforded the title compound (10 mg, 0.040 mmol, 54%) as a pale yellow oil; R_f 0.36 (7 : 3 petrol / EtOAc); IR (thin film, ν_{max} / cm^{-1}) 3448, 2920, 1588, 1500, 1455, 1434, 1310, 1254, 1177, 1083; ^{1}H NMR (400 MHz, $CDCl_3$) δ 7.09 (td, *J* = 7.7, 1.6 Hz, 1H), 7.02 – 6.92 (m, 2H), 6.89 (td, *J* = 7.6, 1.4 Hz, 1H), 6.51 (d, *J* = 1.9 Hz, 1H), 6.42 (d, *J* = 1.7 H, 1H), 5.97 – 5.83 (m, 1H), 5.80 (s, 1H), 5.11 – 4.96 (m, 2H), 3.90 (s, 3H), 3.89 (s, 3H), 3.25 (d, *J* = 6.6 Hz, 2H); ^{13}C NMR (101 MHz, $CDCl_3$) δ 150.6, 148.0, 146.2, 144.2, 137.5, 135.4, 131.3, 124.4, 121.1, 119.4, 115.9, 112.6, 112.3, 107.5, 56.4, 56.1, 40.0; HRMS (ESI+) calc. for $C_{17}H_{18}O_4$ $[M+Na]^+$ 309.1097, found 309.1097.

4-Allyl-2-methoxy-6-(2-methoxyphenoxy)phenyl acetate, 11

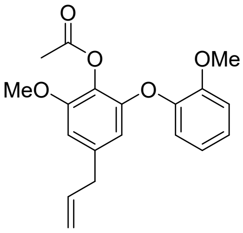

The product was prepared according to General Method G from 4-allyl-2-methoxy-6-(2methoxyphenoxy)phenol, **15** (87 mg, 0.29 mmol). Purification *via* column chromatography (1 : 0 → 4 : 1 petrol / EtOAc) afforded the title compound (59 mg, 0.18 mmol, 61%) as a white solid; MP 74 – 77 °C (pentane); R_f 0.46 (4 : 1 petrol / EtOAc); IR (thin film, ν_{max} / cm^{-1}) 2918, 1762, 1610, 1499, 1437, 1364, 1306, 1256, 1161, 1116, 1094; ^{1}H NMR (400 MHz, $CDCl_3$) δ 7.02 (td, *J* = 8.1, 1.9 Hz, 1H), 6.88 (m, 2H), 6.80 (td, *J* = 7.6, 1.5 Hz, 1H), 6.44 (d, *J* = 1.8 Hz, 1H), 6.21 (d, *J* = 1.8 Hz, 1H), 5.80 (ddt, *J* = 16.0, 10.9, 6.7 Hz, 1H), 5.01 – 4.92 (m, 2H), 3.75 (s, 3H), 3.74 (s, 3H), 3.18 (d, *J* = 6.7 Hz, 2H), 2.15 (s, 3H); ^{13}C NMR (101 MHz, $CDCl_3$) δ 168.6, 152.4, 151.1, 150.0, 145.4, 138.5, 136.8, 131.3, 124.7, 121.1, 120.6, 116.3, 112.9, 110.8, 106.9, 56.2, 56.1, 40.3, 20.4; HRMS (ESI+) calc. for $C_{19}H_{20}O_5$ $[M+H]^+$ 329.1384, found 329.1382.

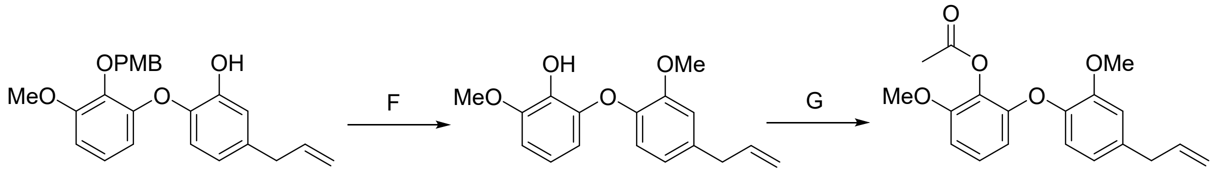

16 12

Scheme 3: *Synthetic routes to analogues **12** and **16**. 1-(4-Allyl-2-methoxyphenoxy)-3-methoxy-2-((4-methoxybenzyl)oxy)benzene prepared as previously published*[2].

2-(4-Allyl-2-methoxyphenoxy)-6-methoxyphenol, 16

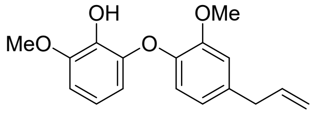

The product was prepared according to General Method F from 1-(4-allyl-2-methoxyphenoxy)-3methoxy-2-((4-methoxybenzyl)oxy)benzene (74 mg, 0.19 mmol). The reaction was complete after 2 h. Purification *via* column chromatography (1 : 0 → 4 : 1 pentane / EtOAc) afforded the title compound (39 mg, 0.14 mmol, 76%) as a colourless oil; R_f 0.33 (4 : 1 pentane / EtOAc); IR (thin film, v_{max} / cm^{-1}) 3502, 2936, 1595, 1505, 1476, 1358, 1261, 1152, 1073, 1033; ^{1}H NMR (400 MHz, $CDCl_3$) δ 6.91 (d, *J* = 8.1 Hz, 1H), 6.80 (s, 1H), 6.78 – 6.68 (m, 2H), 6.66 (d, *J* = 8.2 Hz, 1H), 6.54 (d, *J* = 8.2 Hz, 1H), 6.06 – 5.89 (m, 2H), 5.14 – 5.05 (m, 2H), 3.90 (s, 3H), 3.85 (s, 3H), 3.37 (d, *J* = 6.7 Hz, 2H); ^{13}C NMR (101 MHz, $CDCl_3$) δ 150.6, 148.2, 145.1, 144.1, 137.3, 137.1, 136.8, 120.9, 120.0, 119.1, 116.1, 113.0, 111.6, 106.8, 56.4, 56.1, 40.1; HRMS (ESI+) calc. for $C_{17}H_{18}O_4$ $[M+Na]^+$ 309.1097, found 309.1097.

2-(4-Allyl-2-methoxyphenoxy)-6-methoxyphenyl acetate, 12

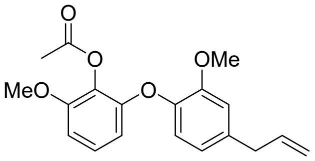

The product was prepared according to General Method G from 2-(4-allyl-2-methoxyphenoxy)-6methoxyphenol, **16** (39 mg, 0.14 mmol). The reaction was complete after 2 h. Purification *via* column chromatography (9 : 1 pentane / EtOAc) afforded the title compound (32 mg, 0.10 mmol, 70%) as an off-white solid; MP 82 – 85 °C; R_f 0.41 (4 : 1 pentane / EtOAc); IR (thin film, v_{max} / cm^{-1}) 2917, 1760, 1505, 1474, 1418, 1251, 1212, 1178, 1083, 1030; ^{1}H NMR (400 MHz, $CDCl_3$) δ 7.03 (td, *J* = 8.3, 1.2 Hz, 1H), 6.89 (d, *J* = 8.1 Hz, 1H), 6.80 (d, *J* = 2.0 Hz, 1H), 6.69 (ddd, *J* = 19.4, 8.3, 1.6 Hz, 2H), 6.40 (dd, *J* = 8.5, 1.4 Hz, 1H), 6.04 – 5.90 (m, 1H), 5.15 – 5.05 (m, 2H), 3.84 (s, 3H), 3.81 (s, 3H), 3.37 (d, *J* = 6.7 Hz, 2H), 2.28 (s, 3H); ^{13}C NMR (101 MHz, $CDCl_3$) δ 168.6, 152.4, 151.1, 150.0, 145.4, 138.5, 136.8, 128.7, 124.7, 121.1, 120.6, 116.3, 112.9, 110.8, 106.9, 56.2, 56.1, 40.3, 20.4; HRMS (ESI+) calc. for $C_{19}H_{20}O_5$ $[M+H]^+$ 329.1384, found 329.1384.

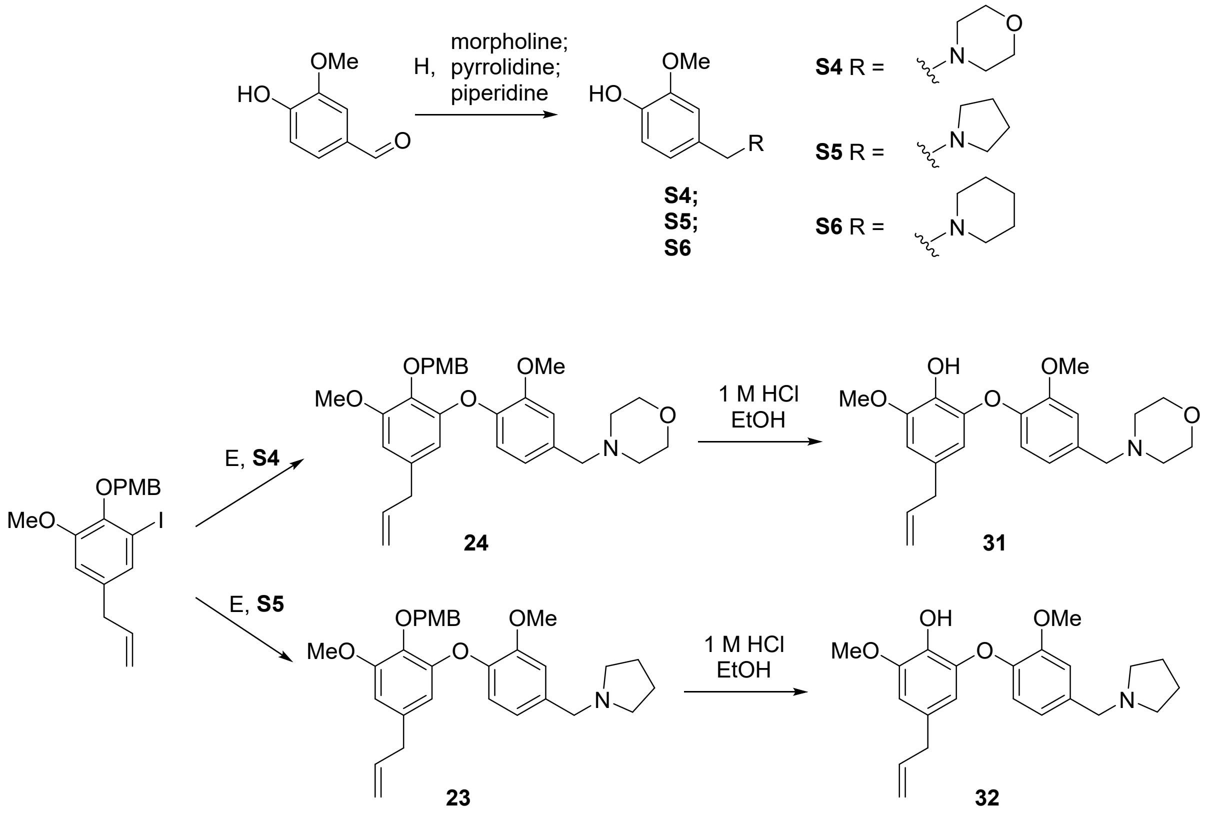

***Scheme 4:** Synthetic routes to analogues **23, 24, 31** and **32**. 5-Allyl-1-iodo-3-methoxy-2-((4-methoxybenzyl)oxy)benzene prepared as previously published*[2].

2-Methoxy-4-(morpholinomethyl)phenol, S4

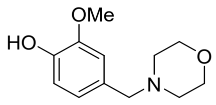

The product was prepared according to General Method H from vanillin (3.00 g, 19.7 mmol) and morpholine (1.71 mL, 19.7 mmol). The reaction was complete after 24 h to afford the title compound (4.28 g, 19.2 mmol, 97%) as a clear gum which was used without any further purification; R_f 0.05 (3 : 2 EtOAc / pentane); ^{1}H NMR (400 MHz, $CDCl_3$) δ 6.79 (d, *J* = 1.9 Hz, 1H), 6.75 (d, *J* = 7.8 Hz, 1H), 6.70 (dd, *J* = 7.8, 1.9 Hz, 1H), 3.78 (s, 3H), 3.66 – 3.62 (m, 4H), 3.35 (s, 2H), 2.40 – 2.33 (m, 4H); ^{13}C NMR (400 MHz, $CDCl_3$) δ 146.7, 145.1, 129.5, 122.4, 114.2, 111.9, 67.1, 63.5, 56.1, 53.7. Spectroscopic data is in accordance with that reported in the literature[3].

2-Methoxy-4-(pyrrolidin-1-ylmethyl)phenol, S5

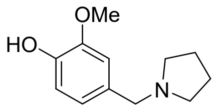

The product was prepared according to General Method H from vanillin (800 mg, 5.26 mmol) and pyrrolidine (0.48 mL, 5.26 mmol). The reaction was complete after 12 h to afford the title compound (750 mg, 3.62 mmol, 69%) as white powder; R_f 0.2 (3 : 2 EtOAc / pentane); ^{1}H NMR (400 MHz, $CDCl_3$) δ 6.93 (br., s, 1H), 6.85 (d, *J* = 7.8 Hz, 1H), 6.80 (d, *J* = 8.0 Hz, 1H), 3.87 (s, 3H), 3.58 (s, 2H), 2.56-2.52

(m, 4H), 1.79-1.73 (m, 4H); ^{13}C NMR (400 MHz, $CDCl_3$) δ 146.9, 145.1, 131.0, 122.0, 114.3, 111.9, 60.8, 56.0, 54.2, 23.6. Spectroscopic data is in accordance with that reported in the literature[3].

2-Methoxy-4-(piperidin-1-ylmethyl)phenol, S6

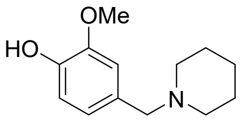

The product was prepared according to General Method H from vanillin (1.00 g, 6.57 mmol) and piperidine (670 µL, 6.57 mmol). The reaction was complete after 16 h. Purification *via* column chromatography (1 : 0 → 3 : 7 pentane / EtOAc) afforded the title compound (0.94 g, 4.2 mmol, 64%) as a white solid; R_f 0.19 (EtOAc); ^1H NMR (400 MHz, $CDCl_3$) δ 6.81 (d, *J* = 1.9 Hz, 1H), 6.72 (d, *J* = 8.0 Hz, 1H), 6.67 (dd, *J* = 8.0, 1.9 Hz, 1H), 3.75 (s, 3H), 3.33 (s, 2H), 2.31 (s, 4H), 1.55 – 1.48 (m, 4H), 1.40 – 1.33 (m, 2H); ^{13}C NMR (101 MHz, $CDCl_3$) δ 146.94, 145.14, 129.17, 129.12, 122.43, 114.28, 63.71, 55.57, 54.32, 25.53, 24.32. Spectroscopic data is in accordance with that reported in the literature[3].

4-(4-(5-Allyl-3-methoxy-2-((4-methoxybenzyl)oxy)phenoxy)-3-methoxybenzyl)morpholine, 24

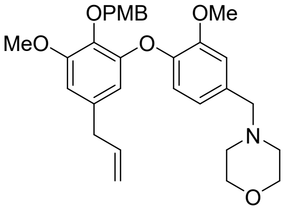

The product was prepared according to General Method E(a) from 5-allyl-1-iodo-3-methoxy-2-((4-methoxybenzyl)oxy)benzene (1.50 g, 3.70 mmol) and 2-methoxy-4-(morpholinomethyl)phenol, **S4** (1.63 g, 7.40 mmol). The reaction was complete after 72 h. Purification *via* column chromatography (1 : 0 → 1 : 1 pentane / EtOAc) afforded the title compound (1.04 g, 2.06 mmol, 56%) as a yellow oil; R_f 0.42 (EtOAc); IR (thin film, v_{max} / cm^{-1}) 2936, 2851, 1585, 1508, 1455, 1420, 1232, 1116, 1091; ^1H NMR (400MHz, $CDCl_3$) δ 7.32 – 7.24 (m, 2H), 6.97 (d, *J* = 1.9 Hz, 1H), 6.82 – 6.77 (m, 2H), 6.76 (dd, *J* = 8.1, 1.9 Hz, 1H), 6.72 (d, *J* = 8.1 Hz, 1H), 6.50 (d, *J* = 2.0 Hz, 1H), 6.36 (d, *J* = 2.0 Hz, 1H), 5.90 (ddt, *J* = 17.5, 9.5, 6.6 Hz, 1H), 5.08 – 5.00 (m, 2H), 4.95 (s, 2H), 3.85 (s, 3H), 3.83 (s, 3H), 3.78 (s, 3H), 3.71 (t, *J* = 4.7 Hz, 4H), 3.45 (s, 2H), 3.26 (dt, *J* = 6.6, 1.5 Hz, 2H), 2.43 (t, *J* = 4.7 Hz, 4H); ^{13}C NMR ($CDCl_3$, 101 MHz) δ 159.2, 154.0, 150.3, 150.2, 145.5, 137.2, 137.1, 135.7, 133.2, 130.1, 130.0, 121.5, 118.2, 115.9, 113.4, 113.3, 112.3, 107.9, 74.6, 67.1, 63.2, 56.1, 56.1, 55.2, 53.6, 40.1, 14.2; HRMS (ESI+) calc. for $C_{30}H_{35}NO_6$ $[M+H]^+$ 506.2532, found 506.2537.

4-Allyl-2-methoxy-6-(2-methoxy-4-(morpholinomethyl)phenoxy)phenol, 31

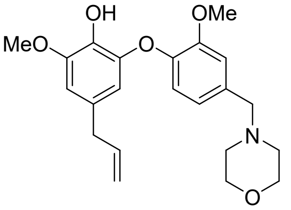

To a solution of 4-(4-(5-allyl-3-methoxy-2-((4-methoxybenzyl)oxy)phenoxy)-3-methoxybenzyl) morpholine, **24** (1.45 g, 2.88 mmol, 1.0 equiv.) in EtOH (100 mL) was added aqueous 1 M HCl (50 mL) and heated to 80 °C while stirring until the reaction was complete (24 h). The solution was basified to pH 7 with aqueous 1 M NaOH, extracted with EtOAc, combined organic extracts washed with brine,

dried over Na_2SO_4 and evaporated to dryness. Purification *via* column chromatography (1:9 to 1:0 EtOAc / pentane) afforded the title compound (758 mg, 1.98 mmol, 69%) as a yellow oil; R_f 0.20 (EtOAc); IR (thin film, v_{max} / cm^{-1}) 3054, 1592, 15122, 1435, 1267, 1189, 1145, 1120, 1085, 736; ^1H NMR (400 MHz, $CDCl_3$) δ 6.98 (d, *J* = 1.9 Hz, 1H), 6.86 (d, *J* = 8.1 Hz, 1H), 6.80 (dd, *J* = 8.1, 1.9 Hz, 1H), 6.50 (d, *J* = 1.8 Hz, 1H), 6.41 (d, *J* = 1.8 Hz, 1H), 5.96 – 5.80 (m, 1H), 5.07 – 4.97 (m, 2H), 3.88 (s, 3H), 3.87 (s, 3H), 3.73 – 3.68 (m, 4H), 3.45 (s, 2H), 3.24 (dt, *J* = 7.0, 1.5 Hz, 2H), 2.47 – 2.40 (m, 4H,; ^{13}C NMR (101 MHz, $CDCl_3$) δ 150.3, 148.0, 145.2, 144.2, 137.4, 135.4, 134.0, 131.1, 121.5, 118.8, 115.7, 113.3, 112.2, 107.4, 67.0, 63.2, 56.2, 56.1, 53.6, 39.9; HRMS (ESI$^+$) calc. for $C_{22}H_{27}NO_5$ $[M+H]^+$ 386.1967, found 386.1963.

1-(4-(5-Allyl-3-methoxy-2-((4-methoxybenzyl)oxy)phenoxy)-3-methoxybenzyl)pyrrolidine, 23

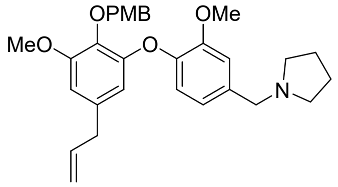

The product was prepared according to General Method E(a) from 5-allyl-1-iodo-3-methoxy-2-((4-methoxybenzyl)oxy)benzene (600 mg, 1.46 mmol) and 2-methoxy-4-(pyrrolidin-1-ylmethyl)phenol, **S5** (454 mg, 2.19 mmol). The reaction was complete after 12 h. Purification *via* column chromatography (1 : 0 → 9 : 1 pentane / Et_2O) afforded the title compound (168 mg, 0.343 mmol, 23%) as a colourless oil; R_f 0.3 (EtOAc); IR (thin film, v_{max} / cm^{-1}) 1586, 1508, 1463, 1421, 1248, 1232, 1212, 1091.61, 996, 918, 731; ^1H NMR (400MHz, $CDCl_3$) δ 7.29 (d, *J* = 8.7 Hz, 2H), 6.99 (d, *J* = 1.5 Hz, 1H), 6.90 (d, *J* = 8.7 Hz, 2H), 6.77-6.72 (m, 2H), 6.48 (d, *J* = 1.8 Hz, 1H), 6.34 (d, 1H, *J* = 1.8Hz), 5.89 (ddt, *J* = 17.5, 9.5, 6.7Hz, 1H), 5.06-5.00 (m, 2H), 4.96 (s, 2H), 3.84 (s, 3H), 3.82 (s, 3H), 3.77 (s, 3H), 3.57 (s, 2H), 3.26 (d, *J* = 6.6 Hz, 2H), 2.51-2.50 (m, 4H), 1.81-1.78 (m, 4H); ^{13}C NMR (101 MHz, $CDCl_3$) δ 159.2, 153.9, 150.5, 150.3, 145.1, 137.1, 137.1, 135.7, 130.2, 130.1, 130.0, 121.2, 118.5, 115.9, 113.4, 113.2, 112.0, 107.7, 74.6, 60.6, 56.1, 56.1, 55.2, 54.2, 40.1, 23.5; HRMS (ESI+) calc. for $C_{30}H_{35}NO_5$ $[M+H]^+$ 490.25836, found 490.25880.

4-Allyl-2-methoxy-6-(2-methoxy-4-(pyrrolidin-1-ylmethyl)phenoxy)phenol, 32

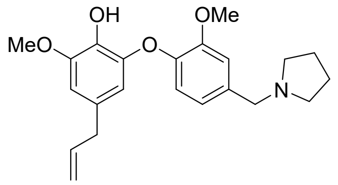

The product was prepared according to General Method F from 1-(4-(5-allyl-3-methoxy-2-((4-methoxybenzyl)oxy)phenoxy)-3-methoxybenzyl)pyrrolidine, **23** (71 mg, 0.15 mmol). The reaction was complete after 3 h. Purification *via* column chromatography pre-washed with 1% triethylamine in petrol (1 : 0 → 1 : 1 petrol / EtOAc) afforded the title compound (15 mg, 0.041 mmol, 27%) as a colourless oil; R_f 0.8 (9 : 1 EtOAc / MeOH); ^1H NMR (400 MHz, $CDCl_3$) δ 6.99 (d, *J* = 1.9 Hz, 1H), 6.88 (d, *J* = 8.1 Hz, 1H), 6.81 (dd, *J* = 8.1, 1.9 Hz, 1H), 6.49 (d, *J* = 1.9 Hz, 1H), 6.40 (d, *J* = 1.9 Hz, 1H), 5.89 (ddt, *J* = 17.9, 9.3, 6.6 Hz, 1H), 5.08 – 4.97 (m, 2H), 3.89 (s, 3H), 3.87 (s, 3H), 3.58 (s, 2H), 3.29 – 3.21 (m, 2H), 2.57 – 2.48 (m, 4H), 1.79 (p, *J* = 3.1 Hz, 4H); ^{13}C NMR (101 MHz, $CDCl_3$) δ 150.3, 147.9, 144.9, 144.4, 137.4, 135.3, 131.0, 121.2, 119.0, 115.7, 113.1, 112.0, 107.3, 60.6, 56.2, 56.0, 54.2, 39.9, 29.7, 23.5; HRMS (ESI+) calc. for $C_{22}H_{27}NO_4$ $[M+H]^+$ 370.2013, found 370.2006.

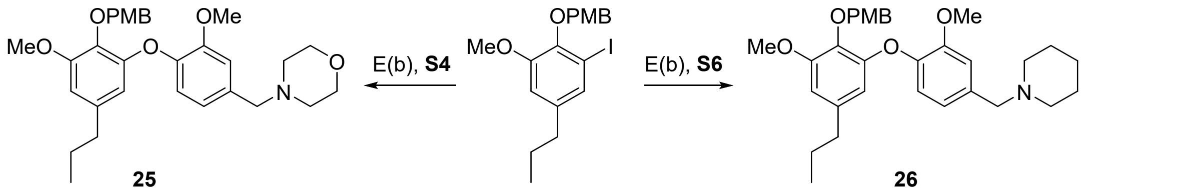

Scheme 5: *Synthetic routes to analogues* ***25*** *and* ***26****. 1-Iodo-3-methoxy-2-((4-methoxybenzyl)oxy)-5-propylbenzene prepared as previously published*[2].

4-(3-Methoxy-4-(3-methoxy-2-((4-methoxybenzyl)oxy)-5-propylphenoxy)benzyl)morpholine, 25

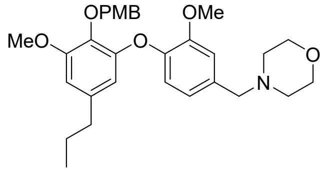

The product was prepared according to General Method E(b) from 1-iodo-3-methoxy-2-((4-methoxybenzyl)oxy)-5-propylbenzene (270 mg, 0.650 mmol) and 2-methoxy-4-(morpholinomethyl)phenol, **S4** (292 mg, 1.31 mmol). The reaction was complete after 48 h. Purification *via* column chromatography pre-washed with 1% triethylamine in pentane (1 : 0 → 3 : 7 pentane / EtOAc) afforded the title compound (220 mg, 0.430 mmol, 66%) as a yellow wax; R_f 0.31 (EtOAc); ^{1}H NMR (400 MHz, $CDCl_3$) δ 7.27 (d, *J* = 8.7 Hz, 2H), 6.98 (s, 1H), 6.78 (d, *J* = 8.7 Hz, 2H), 6.75 (d, *J* = 1.9 Hz, 1H), 6.71 (d, *J* = 8.1 Hz, 1H), 6.50 (d, *J* = 1.9 Hz, 1H), 6.37 (d, *J* = 1.9 Hz, 1H), 4.94 (s, 2H), 3.86 (s, 3H), 3.83 (s, 3H), 3.77 (s, 3H), 3.72 (t, *J* = 4.6 Hz, 4H), 3.46 (s, 2H), 2.50 – 2.40 (m, 6H), 1.63 – 1.52 (m, 2H), 0.89 (t, *J* = 7.3 Hz, 3H); ^{13}C NMR (101 MHz, $CDCl_3$) δ 159.2, 153.8, 150.1, 149.9, 145.9, 138.5, 137.0, 130.2, 130.0, 121.6, 117.8, 113.4, 112.3, 108.0, 74.6, 66.9, 63.2, 56.1, 56.1, 55.2, 53.5, 38.0, 24.4, 13.7; HRMS (ESI+) calc. for $C_{30}H_{37}NO_6$ $[M+H]^+$ 508.2694, found 508.2694.

1-(3-Methoxy-4-(3-methoxy-2-((4-methoxybenzyl)oxy)-5-propylphenoxy)benzyl)piperidine, 26

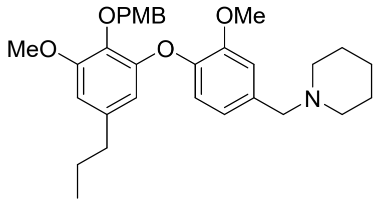

The product was prepared according to General Method E(b) from 1-iodo-3-methoxy-2-((4-methoxybenzyl)oxy)-5-propylbenzene (50 mg, 0.12 mmol) and 2-methoxy-4-(piperidin-1-ylmethyl)phenol, **S6** (54 mg, 0.24 mmol). The reaction was complete after 48 h. Purification *via* column chromatography (1 : 0 → 3 : 7 pentane / EtOAc) afforded the title compound (42 mg, 0.083 mmol, 69%) as a yellow wax; R_f 0.28 (EtOAc); ^{1}H NMR (400 MHz, $CDCl_3$) δ 7.27 (d, *J* = 8.6 Hz, 2H), 7.02 (s, 1H), 6.79 (d, *J* = 8.7 Hz, 2H), 6.75 (dd, *J* = 8.1, 1.9 Hz, 1H), 6.71 (d, *J* = 8.1 Hz, 1H), 6.49 (d, *J* = 2.1 Hz, 1H), 6.36 (d, *J* = 1.9 Hz, 1H), 4.95 (s, 2H), 3.85 (s, 3H), 3.82 (s, 3H), 3.77 (s, 3H), 3.46 (s, 2H), 2.49 – 2.36 (m, 6H), 1.64 – 1.54 (m, 6H), 1.49 – 1.42 (m, 2H), 0.89 (t, *J* = 7.3 Hz, 3H); ^{13}C NMR (101 MHz, $CDCl_3$) δ 158.2, 152.8, 149.1, 144.5, 137.4, 135.9, 133.2, 129.2, 129.0, 121.3, 120.5, 116.9, 112.5, 112.4, 111.1, 106.8, 73.6, 62.4, 55.1, 55.1, 54.2, 53.4, 37.0, 24.8, 23.4, 23.3, 12.7; HRMS (ESI+) calc. for $C_{31}H_{39}NO_5$ $[M+H]^+$ 506.2901, found 506.2903.

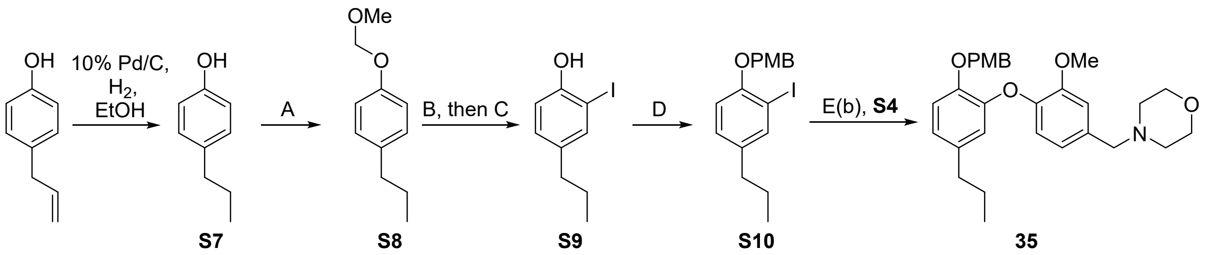

Scheme 6: *Synthetic routes to analogue **35**. 4-Allylphenol prepared as previously published*[2].

4-Propylphenol, S7

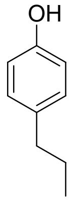

A suspension of 10% w/w Pd/C (0.71 g, 6.7 mmol, 0.3 equiv.) in EtOH (250 mL) was evacuated and backfilled with H_2 (× 3) before addition of 4-allylphenol (3.00 mL, 22.0 mmol, 1.0 equiv.) and a further purge with H_2 (× 3). The resulting suspension was stirred under a H_2 atmosphere until the reaction was complete (2 h), then filtered through a pad of Celite® and washed with EtOAc. The filtrate was dried over Na_2SO_4 and evaporated to dryness to afford the title compound (2.91 g, 21.4 mmol, 96%) as a yellow oil; R_f 0.50 (9 : 1 pentane / EtOAc); ^{1}H NMR (400 MHz, $CDCl_3$) δ 6.97 (d, *J* = 8.6 Hz, 2H), 6.68 (d, *J* = 8.5 Hz, 2H), 4.55 (s, 1H), 2.44 (t, 2H), 1.58 – 1.47 (m, 2H), 0.85 (t, *J* = 7.3 Hz, 3H); ^{13}C NMR (101 MHz, $CDCl_3$) δ 153.4, 135.0, 129.5, 115.0, 37.2, 24.8, 13.8. Spectroscopic data is in accordance with that reported in the literature[4].

1-(Methoxymethoxy)-4-propylbenzene, S8

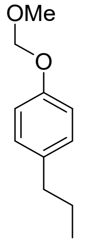

The product was prepared according to General Method A from 4-propylphenol, **S7** (1.50 g, 11.0 mmol). Purification *via* column chromatography (19 : 1 pentane / EtOAc) afforded the title compound (1.15 g, 6.38 mmol, 58%); R_f 0.75 (9 : 1 pentane / EtOAc); ^{1}H NMR (400 MHz, $CDCl_3$) δ 7.09 (d, *J* = 8.7 Hz, 2H), 6.96 (d, *J* = 8.7 Hz, 2H), 5.15 (s, 2H), 3.48 (s, 3H), 2.56 – 2.51 (m, 2H), 1.61 (dq, *J* = 14.8, 7.4 Hz, 2H), 0.93 (t, *J* = 7.3 Hz, 3H); ^{13}C NMR (101 MHz, $CDCl_3$) δ 155.3, 136.2, 129.3, 116.1, 94.7, 55.9, 37.2, 24.7, 13.8. Spectroscopic data is in accordance with that reported in the literature[5].

2-Iodo-4-propylphenol, S9

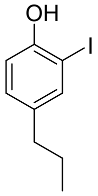

2-Iodo-1-(methoxymethoxy)-4-propylbenzene (900 mg, 3.42 mmol) was freshly synthesized according to General Method B from 1-(methoxymethoxy)-4-propylbenzene, **S8** (1.10 g, 6.11 mmol). This was then used to prepare the product according to General Method C. The reaction was complete after 24 h. Purification *via* column chromatography (1 : 0 → 4 : 1 pentane / EtOAc) afforded the title compound (600 mg, 2.68 mmol, 44%) as a white wax; R_f 0.77 (9 : 1 pentane / EtOAc); ^{1}H NMR (400 MHz, $CDCl_3$) δ 7.46 (d, *J* = 2.1 Hz, 1H), 7.04 (dd, *J* = 8.2, 2.0 Hz, 1H), 6.90 (d, *J* = 8.3 Hz, 1H), 5.10 (s, 1H), 2.52 – 2.44 (m, 2H), 1.65 – 1.53 (m, 2H), 0.92 (t, *J* = 7.3 Hz, 3H); ^{13}C NMR (101 MHz, $CDCl_3$) δ 152.8, 137.8, 137.0, 130.3, 114.7, 85.5, 36.6, 24.6, 13.7. Spectroscopic data is in accordance with that reported in the literature[6].

2-Iodo-1-((4-methoxybenzyl)oxy)-4-propylbenzene, S10

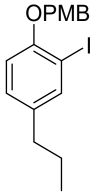

The product was prepared according to General Method D from 2-iodo-4-propylphenol, **S9** (600 mg, 2.30 mmol) and 4-methoxybenzyl chloride (373 µL, 2.75 mmol). The reaction was complete after 2 h. Purification *via* column chromatography (9 : 1 pentane / EtOAc) afforded the title compound (650 mg, 1.70 mmol, 74%) as a white solid; R_f 0.83 (9 : 1 pentane / EtOAc); ^{1}H NMR (400 MHz, $CDCl_3$) δ 7.62 (d, *J* = 2.1 Hz, 1H), 7.42 (d, *J* = 8.8 Hz, 2H), 7.07 (dd, *J* = 8.3, 2.1 Hz, 1H), 6.92 (d, *J* = 8.7 Hz, 2H), 6.78 (d, *J* = 8.3 Hz, 1H), 5.05 (s, 2H), 3.82 (s, 3H), 2.53 – 2.44 (m, 2H), 1.65 – 1.53 (m, 2H), 0.92 (t, *J* = 7.3 Hz, 3H); ^{13}C NMR (101 MHz, $CDCl_3$) δ 159.3, 155.4, 139.3, 137.3, 129.3, 128.8, 128.7, 113.9, 112.9, 86.9, 71.0, 55.3, 36.7, 24.6, 13.7; HRMS (ESI+) calc. for $C_{17}H_{19}IO_2$ $[M+H]^+$ 383.0503, found 383.0497.

4-(3-Methoxy-4-(2-((4-methoxybenzyl)oxy)-5-propylphenoxy)benzyl)morpholine, 35

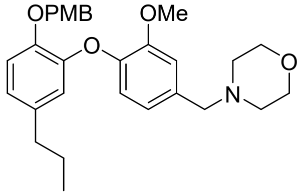

The product was prepared according to General Method E(b) from 2-iodo-1-((4-methoxybenzyl)oxy)-4-propylbenzene, **S10** (20 mg, 0.052 mmol) and 2-methoxy-4-(morpholinomethyl)phenol, **S4** (23 mg, 0.10 mmol). The reaction was complete after 48 h. Purification *via* column chromatography pre-washed with 1% triethylamine in petrol (1 : 0 → 3 : 7 petrol / EtOAc) afforded the title compound (17 mg, 0.035 mmol, 69%) as a yellow wax; R_f 0.35 (EtOAc); ^{1}H NMR (400 MHz, $CDCl_3$) δ 7.16 – 7.10 (m, 2H), 6.99 (s, 1H), 6.90 (d, *J* = 8.2 Hz, 1H), 6.86 – 6.73 (m, 5H), 6.66 (d, *J* = 8.1 Hz, 1H), 4.98 (s, 2H), 3.88 (s, 3H), 3.78 (s, 3H), 3.73 (s, 4H), 3.48 (s, 2H), 2.53 – 2.43 (m, 6H), 1.63 – 1.49 (m, 2H), 0.89 (t, *J* = 7.3 Hz, 3H); ^{13}C NMR (101 MHz, $CDCl_3$) δ 159.1, 149.9, 148.0, 136.4, 129.4, 128.8, 128.7, 124.6, 124.3, 121.1, 116.8, 115.7, 113.7, 113.6, 111.4, 105.5, 71.0, 66.4, 63.1, 56.3, 55.3, 53.4, 37.2, 24.5, 13.7; HRMS (ESI+) calc. for $C_{29}H_{35}NO_5$ $[M+H]^+$ 478.2558, found 478.2586.

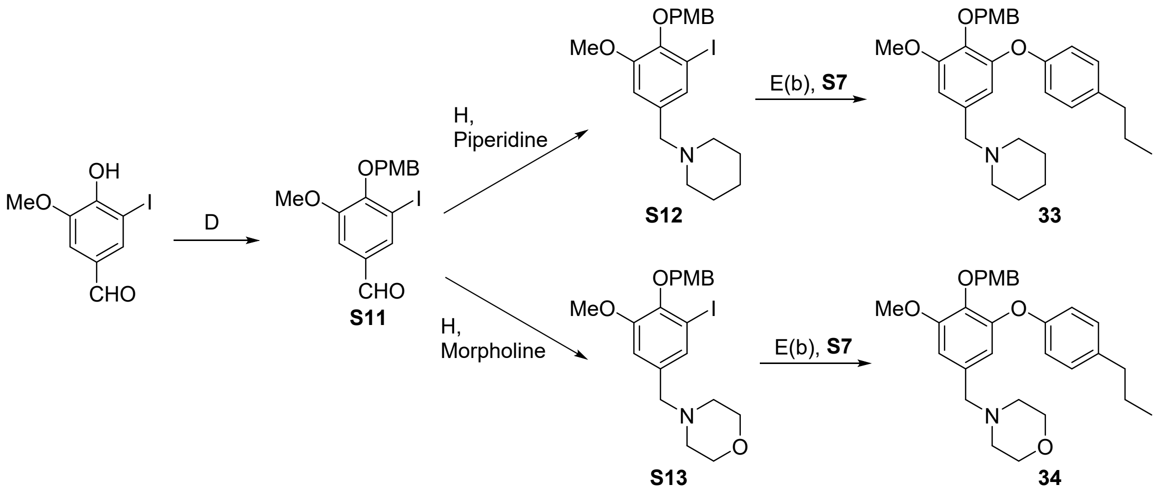

Scheme 7: *Synthetic routes to analogues* ***33*** *and* ***34*** *from 5-iodovanillin.*

3-Iodo-5-methoxy-4-((4-methoxybenzyl)oxy)benzaldehyde, S11

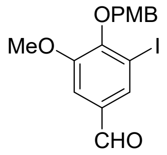

The product was prepared according to General Method D from 5-iodovanillin (1.00 g, 3.60 mmol) and 4-methoxybenzyl chloride (585 µL, 4.31 mmol). The reaction was complete after 2 h. Purification *via* column chromatography (1 : 0 → 9 : 1 pentane / EtOAc) afforded the title compound (1.23 g, 3.09 mmol, 87%) as a white solid; R_f 0.16 (9 : 1 pentane / EtOAc); ^{1}H NMR (400 MHz, $CDCl_3$) δ 9.82 (s, 1H), 7.84 (d, *J* = 1.8 Hz, 1H), 7.46 (d, *J* = 8.7 Hz, 2H), 7.41 (d, *J* = 1.8 Hz, 1H), 6.90 (d, *J* = 8.7 Hz, 2H), 5.09 (s, 2H), 3.94 (s, 3H), 3.81 (s, 3H); ^{13}C NMR (101 MHz, $CDCl_3$) δ 189.7, 159.8, 153.1, 153.0, 134.9, 133.9, 130.5, 128.6, 113.8, 111.0, 93.0, 74.6, 56.1, 55.3; HRMS (ESI+) calc. for $C_{16}H_{15}IO_4$ $[M+H]^+$ 399.0088, found 399.0089.

1-(3-Iodo-5-methoxy-4-((4-methoxybenzyl)oxy)benzyl)piperidine, S12

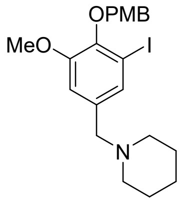

The product was prepared according to General Method H from 3-iodo-5-methoxy-4-((4-methoxybenzyl)oxy)benzaldehyde, **S11** (0.10 g, 0.25 mmol) and piperidine (26 µL, 0.25 mmol). The reaction was complete after 16 h. Purification *via* column chromatography pre-washed with 1% triethylamine in pentane (1 : 0 → 3 : 7 pentane / EtOAc) afforded the title compound (56 mg, 0.12 mmol, 48%) as a yellow wax; R_f 0.10 (EtOAc); ^{1}H NMR (400 MHz, $CDCl_3$) δ 7.50 (d, *J* = 8.7 Hz, 2H), 7.28 (d, *J* = 1.9 Hz, 1H), 7.26 (s, 1H), 6.91 (d, *J* = 8.7 Hz, 2H), 4.93 (s, 2H), 3.88 (s, 3H), 3.82 (s, 3H), 3.39 (s, 2H), 2.37 (s, 4H), 1.61 (s, 4H), 1.45 (s, 2H); ^{13}C NMR (101 MHz, $CDCl_3$) δ 159.5, 152.6, 146.6, 137.0, 130.8, 130.3, 129.5, 113.7, 113.6, 92.7, 74.2, 62.9, 56.1, 55.3, 54.5, 26.0, 24.3; HRMS (ESI+) calc. for $C_{21}H_{26}INO_3$ $[M+H]^+$ 468.1030, found 468.1021.

4-(3-Iodo-5-methoxy-4-((4-methoxybenzyl)oxy)benzyl)morpholine, S13

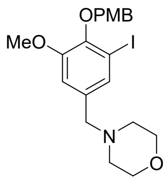

The product was prepared according to General Method H from 3-iodo-5-methoxy-4-((4-methoxybenzyl)oxy)benzaldehyde, **S11** (0.10 mg, 0.25 mmol) and morpholine (22 µL, 0.25 mmol). The reaction was complete after 16 h. Purification *via* column chromatography pre-washed with 1% triethylamine in pentane (1 : 0 → 3 : 7 pentane / EtOAc) afforded the title compound (82 mg, 0.17 mmol, 70%) as a white solid; R_f 0.34 (EtOAc); IR (thin film, ν_{max} / cm^{-1}) 2997, 2955, 2909, 2834, 2808, 2361, 1612, 1587, 1561, 1513, 1460, 1410, 1371, 1348, 1331, 1302, 1267, 1248, 1221, 1174, 1143, 1115, 1069, 1041, 1008; ^{1}H NMR (400 MHz, $CDCl_3$) δ 7.50 (d, *J* = 8.7 Hz, 2H), 7.30 (d, *J* = 1.8 Hz, 1H), 6.91 (d, *J* = 8.7 Hz, 3H), 4.93 (s, 2H), 3.88 (s, 3H), 3.82 (s, 3H), 3.74 (s, 4H), 3.42 (s, 2H), 2.45 (s, 4H); ^{13}C NMR (101 MHz, $CDCl_3$) δ 159.6, 152.7, 146.9, 136.0, 130.8, 130.3, 129.4, 113.7, 113.6, 92.8, 74.3, 67.0, 62.5, 56.1, 55.3, 53.6; HRMS (ESI+) calc. for $C_{20}H_{24}INO_4$ $[M+H]^+$ 470.0823, found 470.0819.

1-(3-Methoxy-4-((4-methoxybenzyl)oxy)-5-(4-propylphenoxy)benzyl)piperidine, 33

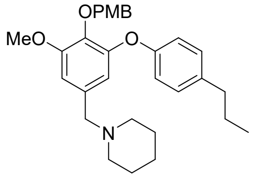

The product was prepared according to General Method E(b) from 1-(3-iodo-5-methoxy-4-((4-methoxybenzyl)oxy)benzyl)piperidine, **S12** (30 mg, 0.060 mmol) and 4-propylphenol, **S7** (17 mg, 0.13 mmol). The reaction was complete after 48 h. Purification *via* column chromatography pre-washed with 1% triethylamine in pentane (1 : 0 → 3 : 7 pentane / EtOAc) afforded the title compound (17 mg, 0.035 mmol, 60%) as a pale-yellow wax; R_f 0.25 (EtOAc); ^{1}H NMR (400 MHz, $CDCl_3$) δ 7.23 (d, *J* = 8.6 Hz, 2H), 7.08 (d, *J* = 8.5 Hz, 2H), 6.83 (d, *J* = 8.6 Hz, 2H), 6.78 (d, *J* = 8.7 Hz, 2H), 6.74 (s, 1H), 6.54 (d, *J* = 1.8 Hz, 1H), 4.93 (s, 2H), 3.87 (s, 3H), 3.77 (s, 3H), 3.37 (s, 2H), 2.57 – 2.52 (m, 2H), 2.35 (s, 4H), 1.68 – 1.54 (m, 6H), 1.42 (s, 2H), 0.94 (t, J = 7.3 Hz, 3H); ^{13}C NMR (101 MHz, $CDCl_3$) δ 159.3, 155.9, 154.0, 149.6, 136.7, 130.1, 129.9, 129.3, 117.2, 113.9, 113.5, 108.5, 74.7, 63.5, 56.2, 55.2, 54.4, 37.3, 25.9, 24.7, 24.3, 13.8; HRMS (ESI+) calc. for $C_{30}H_{37}NO_4$ $[M+H]^+$ 476.2795, found 476.2795.

4-(3-Methoxy-4-((4-methoxybenzyl)oxy)-5-(4-propylphenoxy)benzyl)morpholine, 34

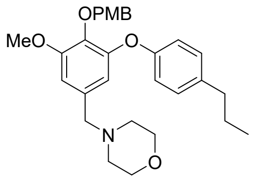

The product was prepared according to General Method E(b) from 4-(3-iodo-5-methoxy-4-((4-methoxybenzyl)oxy)benzyl)morpholine, **S13** (50 mg, 0.11 mmol) and 4-propylphenol, **S7** (29 µL, 0.21 mmol). The reaction was complete after 48 h. Purification *via* column chromatography pre-washed with 1% triethylamine in pentane (1 : 0 → 3 : 7 pentane / EtOAc) afforded the title compound (30 mg, 0.062 mmol, 59%) as a yellow wax; R_f 0.21 (1 : 1 pentane / EtOAc); ^{1}H NMR (400 MHz, $CDCl_3$) δ 7.23 (d, *J* = 8.7 Hz, 2H), 7.08 (d, *J* = 8.5 Hz, 2H), 6.83 (d, *J* = 8.5 Hz, 2H), 6.79 (d, *J* = 8.7 Hz, 2H), 6.72 (s, 1H), 6.56 (d, *J* = 1.9 Hz, 1H), 4.93 (s, 2H), 3.87 (s, 3H), 3.77 (s, 3H), 3.69 (t, *J* = 4.6 Hz, 4H), 3.39 (s, 2H), 2.57 – 2.53 (m, 2H), 2.45 – 2.38 (m, 4H), 1.68 – 1.58 (m, 2H), 0.94 (t, *J* = 7.3 Hz, 3H);

^{13}C NMR (101 MHz, $CDCl_3$) δ 159.3, 155.8, 154.1, 149.8, 138.6, 136.8, 130.1, 129.8, 129.4, 129.0, 117.2, 113.8, 113.5, 108.4, 74.7, 67.0, 63.1, 56.2, 55.2, 53.5, 37.3, 24.7, 13.8; HRMS (ESI+) calc. for $C_{29}H_{35}NO_5$ $[M+H]^+$ 478.2588, found 478.2589.

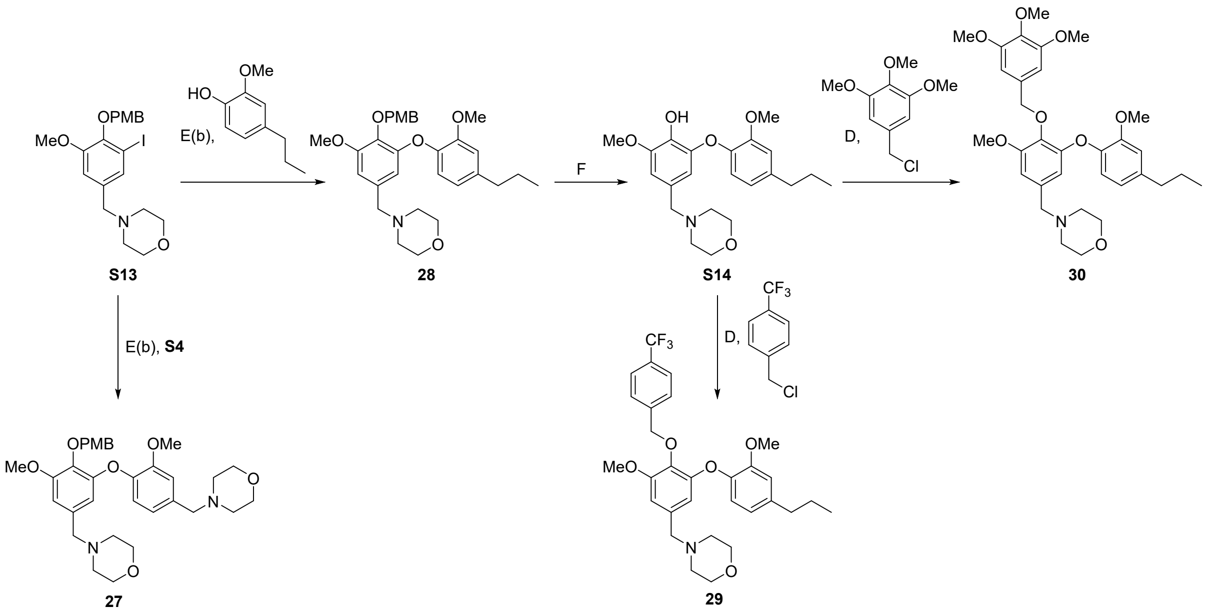

Scheme 8: *Synthetic routes to analogues **27** – **30**. 2-Methoxy-4-propylphenol was prepared as previously published*[2].

4-(3-Methoxy-4-(3-methoxy-2-((4-methoxybenzyl)oxy)-5-(morpholinomethyl)phenoxy)benzyl) morpholine, 27

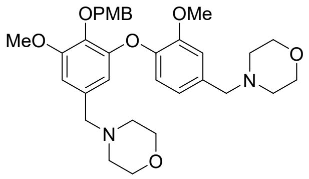

The product was prepared according to General Method E(b) from 4-(3-iodo-5-methoxy-4-((4-methoxybenzyl)oxy)benzyl)morpholine, **S13** (80 mg, 0.17 mmol) and 2-methoxy-4-(morpholinomethyl)phenol, **S4** (60 mg, 0.26 mmol). The reaction was complete after 96 h. Purification *via* column chromatography pre-washed with 1% triethylamine in EtOAc (1 : 0 → 9 : 1 EtOAc / MeOH) afforded the title compound (10 mg, 0.018 mmol, 11%) as a colourless oil; R_f 0.2 (19 : 1 EtOAc / MeOH); IR (thin film, ν_{max} / cm^{-1}) 2956, 2807, 2764, 1710, 1610, 1587, 1454, 1423, 1350, 1268, 1249, 1219, 1116, 1092, 1035; ^1H NMR (400 MHz, $CDCl_3$) δ 7.27 (dd, *J* = 6.9, 1.8 Hz, 2H), 6.98 (s, 1H), 6.78 (dd, *J* = 9.2, 2.5 Hz, 2H), 6.75 (d, *J* = 1.9 Hz, 1H), 6.70 (d, *J* = 8.1 Hz, 2H), 6.51 (d, *J* = 1.8 Hz, 1H), 4.96 (s, 2H), 3.85 (s, 3H), 3.84 (s, 3H), 3.77 (s, 3H), 3.71 (d, *J* = 4.7 Hz, 4H), 3.68 (t, *J* = 4.6 Hz, 4H), 3.47 (s, 2H), 3.38 (s, 2H), 2.45 (s, 4H), 2.40 (s, 4H); ^{13}C NMR (126 MHz, $CDCl_3$) δ 159.3, 154.1, 150.1, 150.0, 150.0, 145.6, 138.0, 130.0, 130.0, 121.6, 117.9, 113.6, 113.4, 113.3, 112.9, 108.2, 74.6, 67.0, 66.9, 63.2, 63.1, 56.2, 56.0, 55.3, 53.5, 53.5; HRMS (ESI+) calc. for $C_{32}H_{40}N_2O_7$ $[M+H]^+$ 565.2908, found 565.2907.

4-(3-Methoxy-5-(2-methoxy-4-propylphenoxy)-4-((4-methoxybenzyl)oxy)benzyl)morpholine, 28

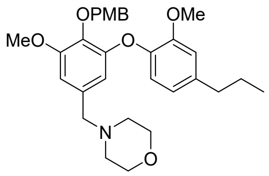

The product was prepared according to General Method E(b) from 4-(3-iodo-5-methoxy-4-((4-methoxybenzyl)oxy)benzyl)morpholine, **S13** (350 mg, 0.745 mmol) and 2-methoxy-4-propylphenol (238 µL, 1.49 mmol). The reaction was complete after 48 h. Purification *via* column chromatography pre-washed with 1% triethylamine in pentane (1 : 0 → 1 : 1 pentane / EtOAc) afforded the title compound (292 mg, 0.575 mmol, 77%) as a colourless oil; R_f 0.36 (EtOAc); IR (thin film, ν_{max} / cm^{-1}) 2957, 2934, 2360, 2341, 1610, 1587, 1510, 1454, 1423, 1266, 1248, 1216, 1117, 1092, 1035, 982; ^{1}H NMR (400 MHz, $CDCl_3$) δ 7.34 – 7.26 (m, 2H), 6.83 – 6.76 (m, 3H), 6.72 (d, *J* = 8.1 Hz, 1H), 6.66 (dd, *J* = 8.1, 1.9 Hz, 2H), 6.45 (d, *J* = 1.9 Hz, 1H), 4.99 (s, 2H), 3.84 (s, 3H), 3.81 (s, 3H), 3.78 (s, 3H), 3.67 (t, *J* = 4.6 Hz, 4H), 3.36 (s, 2H), 2.56 (t, *J* = 7.3 Hz, 2H), 2.39 (t, *J* = 4.7 Hz, 4H), 1.72 – 1.58 (m, 2H), 0.96 (t, *J* = 7.3 Hz, 3H); ^{13}C NMR (101 MHz, $CDCl_3$) δ 159.3, 154.0, 150.6, 150.2, 144.0, 138.5, 137.7, 133.4, 130.2, 130.1, 120.6, 118.9, 113.4, 113.0, 112.2, 107.7, 74.6, 67.0, 63.1, 56.2, 55.9, 55.2, 53.5, 37.8, 24.7, 13.8; HRMS (ESI+) calc. for $C_{30}H_{37}NO_6$ $[M+H]^+$ 508.2694, found 508.2695.

2-Methoxy-6-(2-methoxy-4-propylphenoxy)-4-(morpholinomethyl)phenol, S14

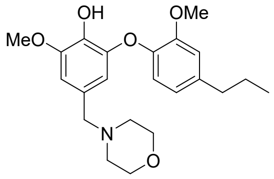

The product was prepared according to General Method F from 4-(3-methoxy-5-(2-methoxy-4-propylphenoxy)-4-((4-methoxybenzyl)oxy)benzyl)morpholine **28** (140 mg, 0.276 mmol). The reaction was complete after 4 h. Purification *via* column chromatography pre-washed with 1% triethylamine in pentane (4 : 1 → 0 : 1 pentane / EtOAc) afforded the title compound (46 mg, 0.12 mmol, 43%); R_f 0.18 (EtOAc); IR (thin film, ν_{max} / cm^{-1}) 2961, 2922, 2361, 2341, 1608, 1595, 1510, 1457, 1432, 1265, 1213, 1155, 1116, 1087, 866; ^{1}H NMR (400 MHz, $CDCl_3$) δ 6.86 (d, *J* = 8.1 Hz, 1H), 6.78 (d, *J* = 2.0 Hz, 1H), 6.68 (dd, *J* = 10.7, 2.0 Hz, 2H), 6.53 (d, *J* = 1.8 Hz, 1H), 3.89 (s, 3H), 3.85 (s, 3H), 3.70 – 3.63 (m, 4H), 3.34 (s, 2H), 2.56 (dd, *J* = 8.5, 6.8 Hz, 2H), 2.41 – 2.32 (m, 4H), 1.71 – 1.57 (m, 2H), 0.95 (t, *J* = 7.3 Hz, 3H); ^{13}C NMR (101 MHz, $CDCl_3$) δ 152.3, 147.9, 143.9, 139.3, 136.0, 128.8, 120.7, 119.4, 112.9, 112.5, 111.6, 107.6, 67.0, 63.2, 56.3, 56.0, 53.5, 37.8, 24.6, 13.8; HRMS (ESI+) calc. for $C_{22}H_{29}NO_5$ $[M+H]^+$ 388.2118, found 388.2119.

4-(3-Methoxy-5-(2-methoxy-4-propylphenoxy)-4-((3,4,5-trimethoxybenzyl)oxy)benzyl)morpholine, 30

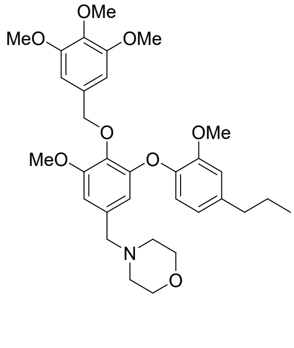

The product was prepared according to General Method D from 2-methoxy-6-(2-methoxy-4-propylphenoxy)-4-(morpholinomethyl)phenol, **S14** (20 mg, 0.052 mmol) and 3,4,5-trimethoxybenzyl chloride (13 mg, 0.060 mmol). The reaction was complete after 18 h. Purification *via* column chromatography pre-washed with 1% triethylamine in pentane (1 : 0 → 2 : 3 pentane / EtOAc) afforded the title compound (18 mg, 0.033 mmol, 61%) as a colourless oil; R_f 0.15 (EtOAc); ^{1}H NMR (400 MHz, $CDCl_3$) δ 6.77 (d, *J* = 1.9 Hz, 1H), 6.73 (d, *J* = 8.1 Hz, 1H), 6.67 (d, *J* = 3.2 Hz, 4H), 6.46 (d, *J* = 1.8 Hz, 1H), 5.02 (s, 2H), 3.87 (s, 3H), 3.80 (s, 3H), 3.79 (s, 3H), 3.76 (s, 6H), 3.67 (t, *J* = 4.6 Hz, 4H), 3.36 (s, 2H), 2.55 (dd, *J* = 8.6, 6.7 Hz, 2H), 2.38 (t, *J* = 4.7 Hz, 4H), 1.70 – 1.57 (m, 2H), 0.95 (t, *J* = 7.3 Hz, 3H); ^{13}C NMR (101 MHz, $CDCl_3$) δ 153.8, 152.9, 150.5, 150.1, 147.0, 143.9, 138.6, 137.9, 137.4, 133.7, 120.6, 118.8, 112.8, 112.3, 107.7, 105.3, 75.3, 67.0, 63.1, 60.8, 56.2, 55.9, 55.9, 53.5, 37.8, 24.7, 13.8; HRMS (ESI+) calc. for $C_{32}H_{41}NO_8$ $[M+H]^+$ 568.2905, found 568.2900.

4-(3-Methoxy-5-(2-methoxy-4-propylphenoxy)-4-((4-(trifluoromethyl)benzyl)oxy)benzyl) morpholine, 29

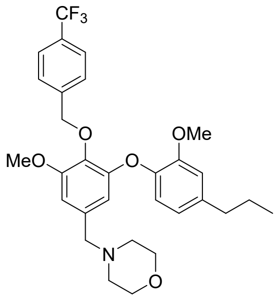

The product was prepared according to General Method D from 2-methoxy-6-(2-methoxy-4-propylphenoxy)-4-(morpholinomethyl)phenol, **S14** (20 mg, 0.052 mmol) and 4-(trifluoromethyl)benzyl chloride (9 µL, 0.060 mmol). The reaction was complete after 18 h. Purification *via* column chromatography pre-washed with 1% triethylamine in pentane (1 : 0 → 3 : 1 pentane / EtOAc) afforded the title compound (13 mg, 0.024 mmol, 46%) as a colourless oil; R_f 0.29 (EtOAc); ^{1}H NMR (400 MHz, $CDCl_3$) δ 7.58 – 7.44 (m, 4H), 6.80 – 6.62 (m, 4H), 6.48 (d, *J* = 1.9 Hz, 1H), 5.11 (s, 2H), 3.85 (s, 3H), 3.77 (s, 3H), 3.67 (t, *J* = 4.6 Hz, 4H), 3.37 (s, 2H), 2.56 (dd, *J* = 8.5, 6.7 Hz, 2H), 2.39 (t, *J* = 4.7 Hz, 4H), 1.71 – 1.56 (m, 2H), 0.95 (t, *J* = 7.3 Hz, 3H); ^{13}C NMR (101 MHz, $CDCl_3$) δ 153.7, 150.4, 150.1, 143.9, 142.1, 138.7, 137.5, 133.9, 128.0, 127.2, 124.9, 120.6, 118.6, 112.8, 112.3, 107.7, 74.0, 67.0, 63.1, 56.1, 55.8, 53.5, 37.8, 24.7, 13.8; HRMS (ESI+) calc. for $C_{30}H_{34}F_3NO_5$ $[M+H]^+$ 546.2462, found 546.2455.

5. Copies of 1H NMR spectra

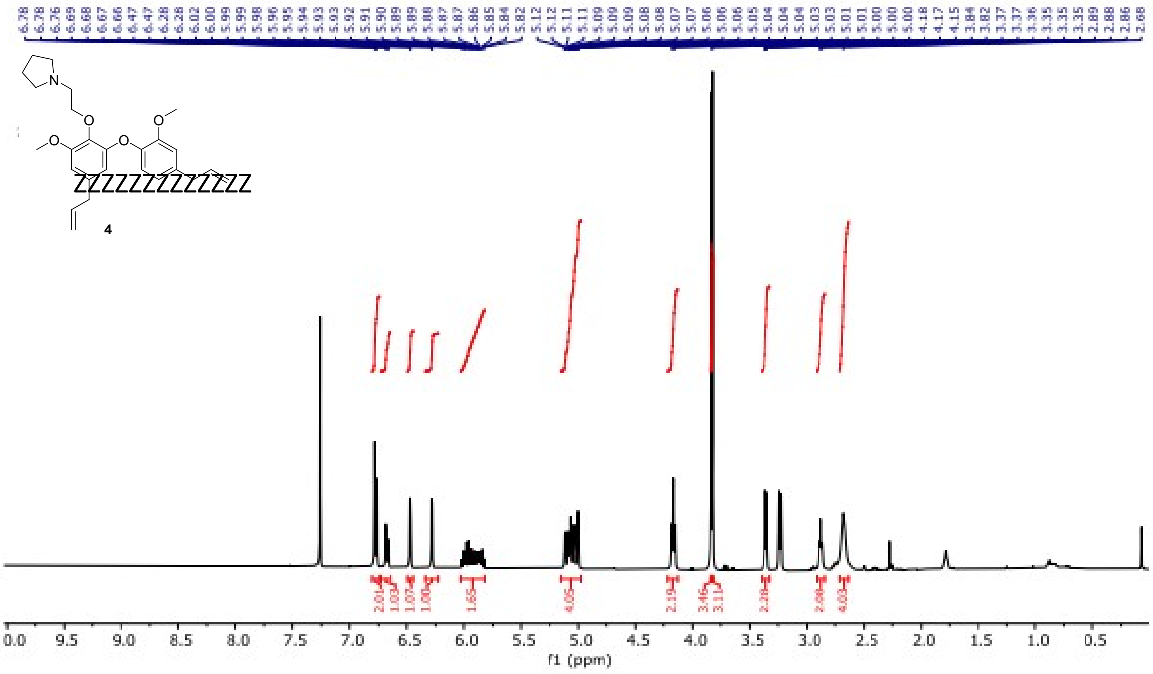

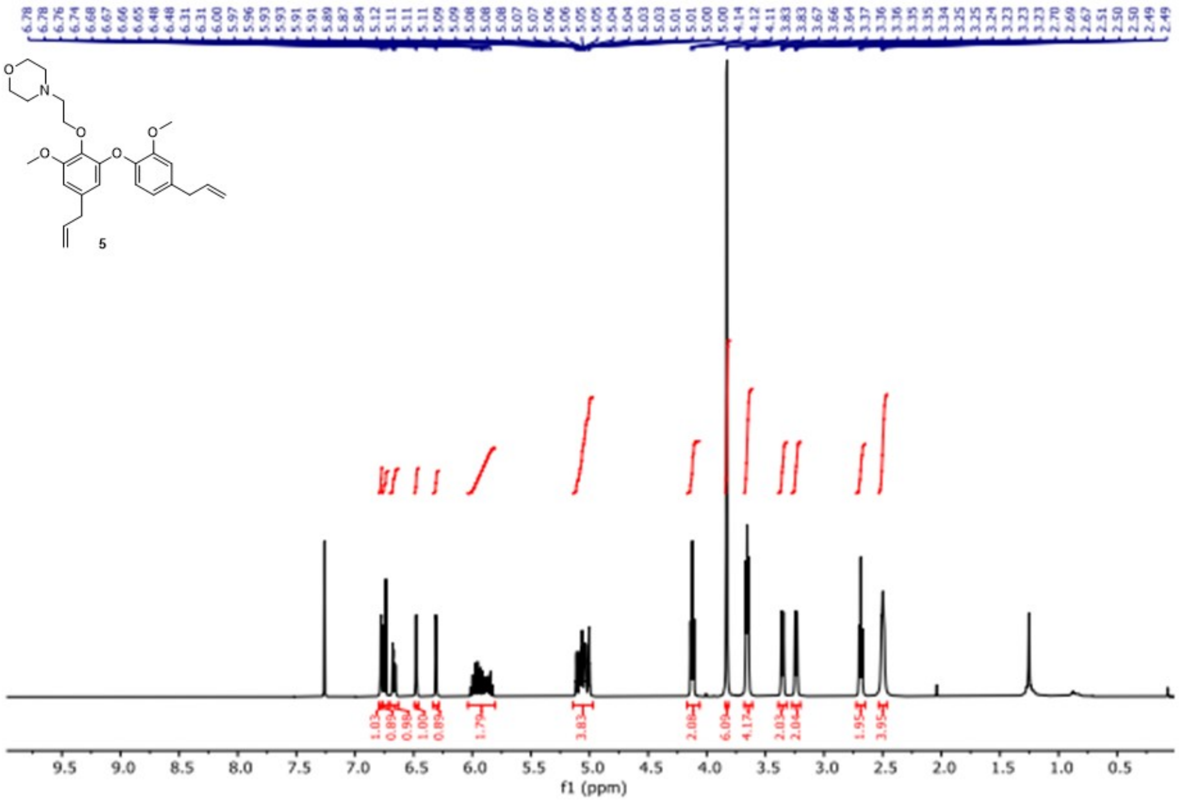

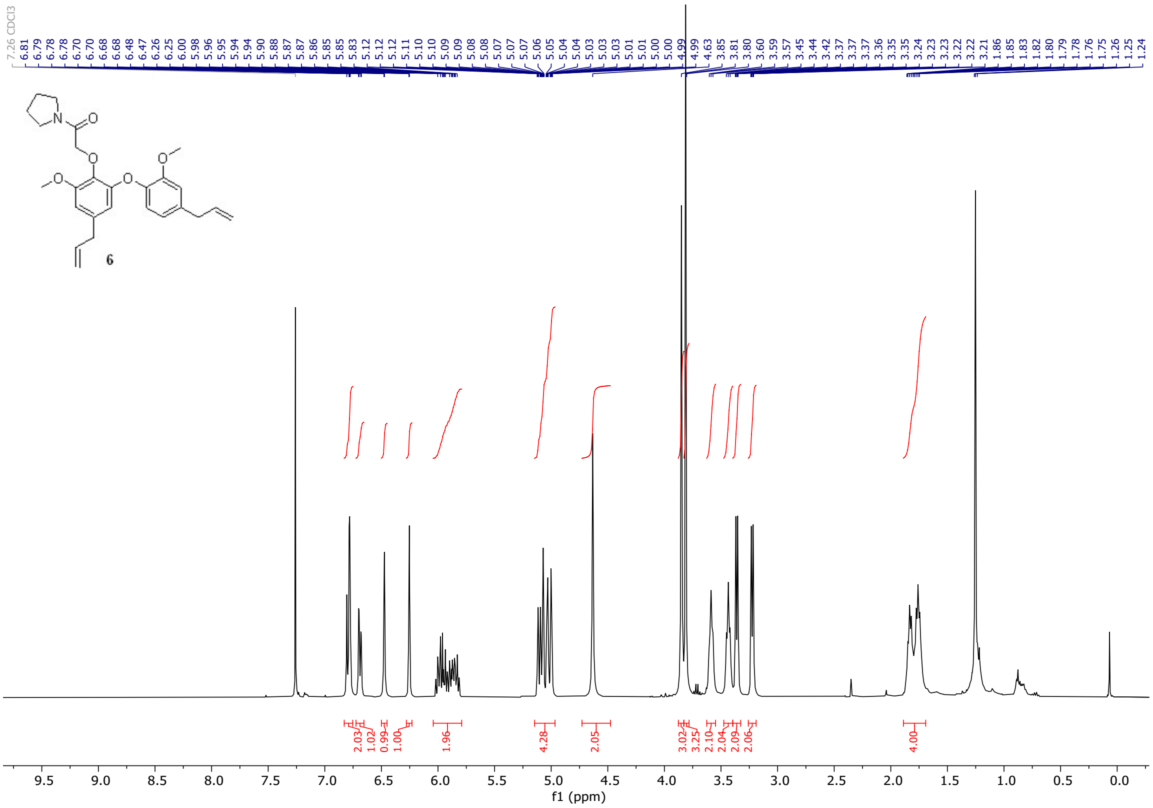
6
f1 (ppm)
9.5 9.0 8.5 8.0 7.5 7.0 6.5 6.0 5.5 5.0 4.5 4.0 3.5 3.0 2.5 2.0 1.5 1.0 0.5 0.0

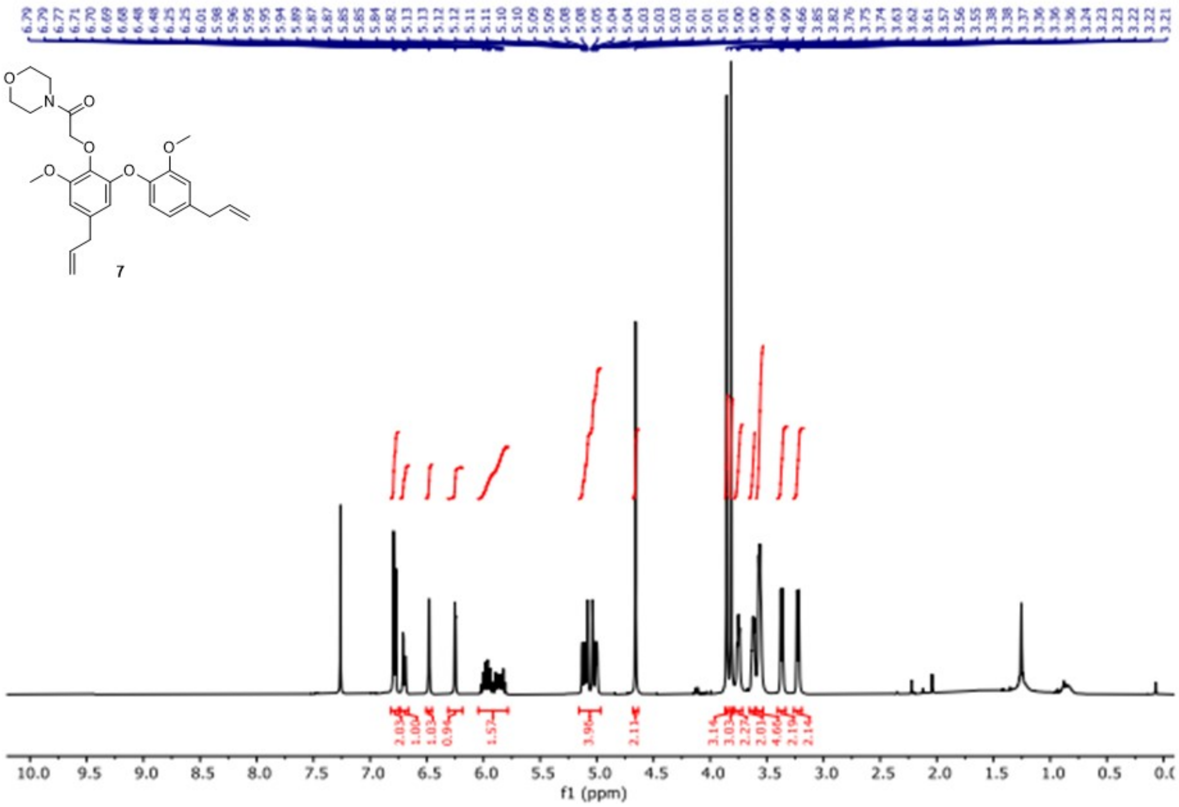
7
f1 (ppm)
10.0 9.5 9.0 8.5 8.0 7.5 7.0 6.5 6.0 5.5 5.0 4.5 4.0 3.5 3.0 2.5 2.0 1.5 1.0 0.5

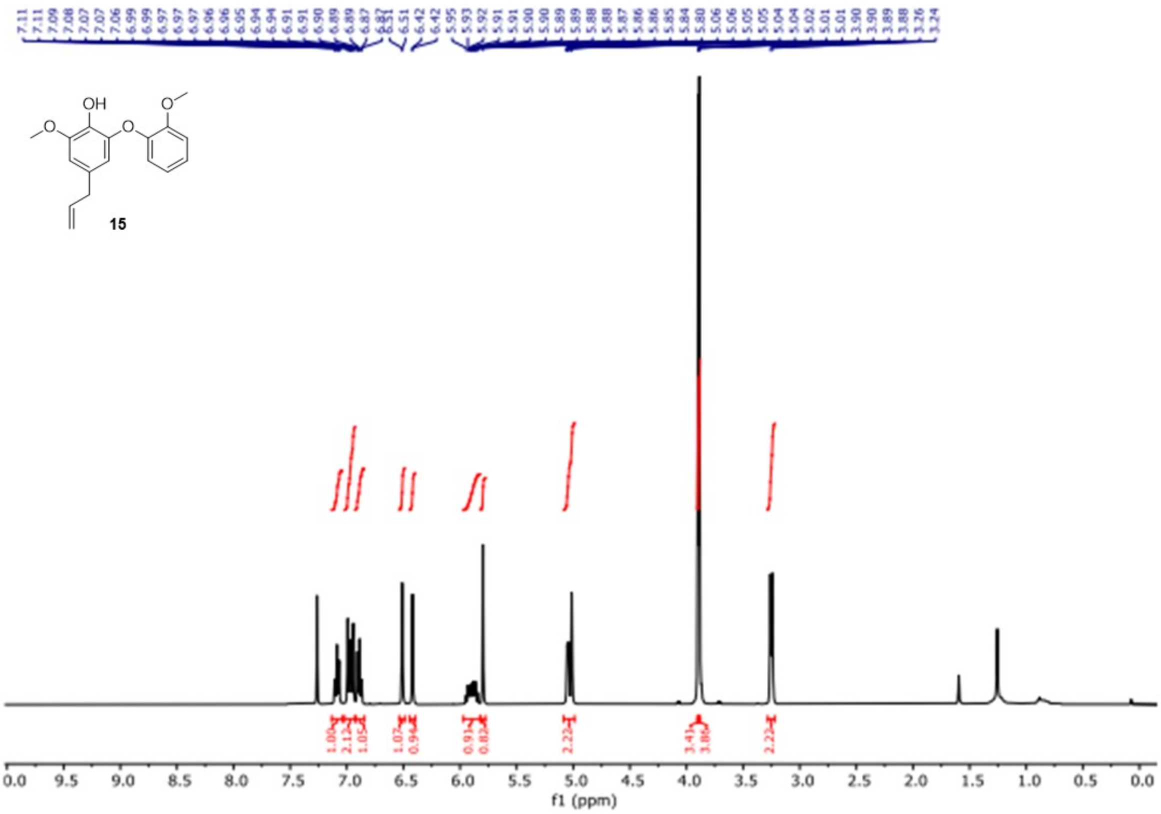
OH
15
f1 (ppm)

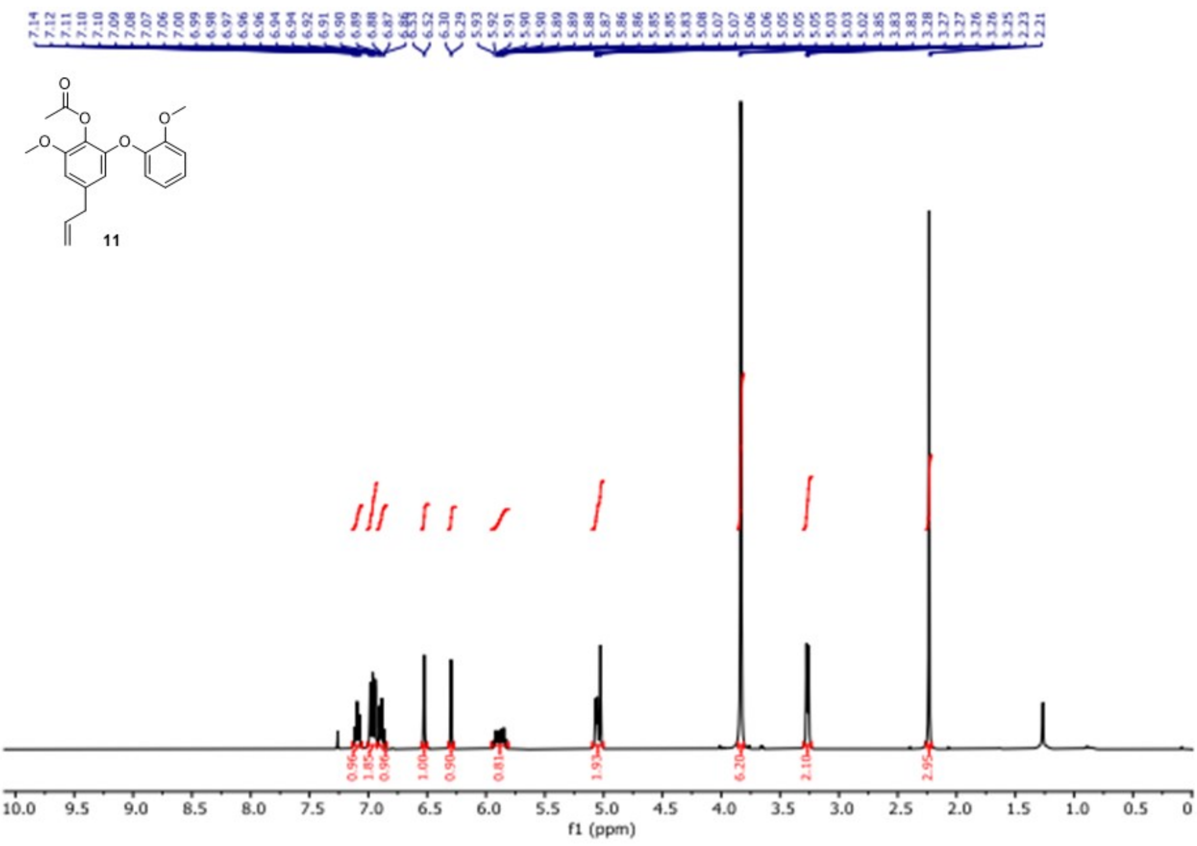
11
f1 (ppm)

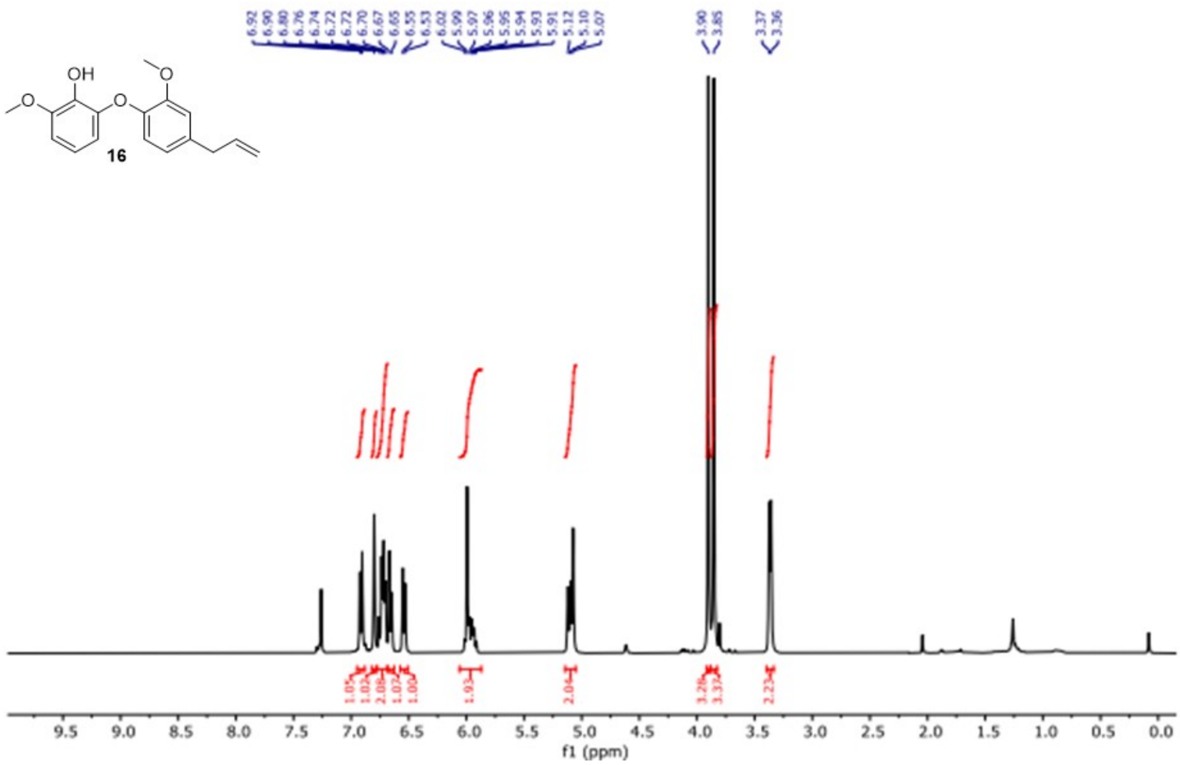
6.92 6.90 6.80 6.76 6.74 6.72 6.72 6.70 6.67 6.65 6.55 6.53 6.02 5.99 5.97 5.96 5.95 5.94 5.93 5.91 5.12 5.10 5.07
3.90 3.85
3.37 3.36
OH
16
1.07 1.02 2.08 1.07 1.00 1.93 2.04 3.28 3.37 2.23
9.5 9.0 8.5 8.0 7.5 7.0 6.5 6.0 5.5 5.0 4.5 4.0 3.5 3.0 2.5 2.0 1.5 1.0 0.5 0.0
f1 (ppm)

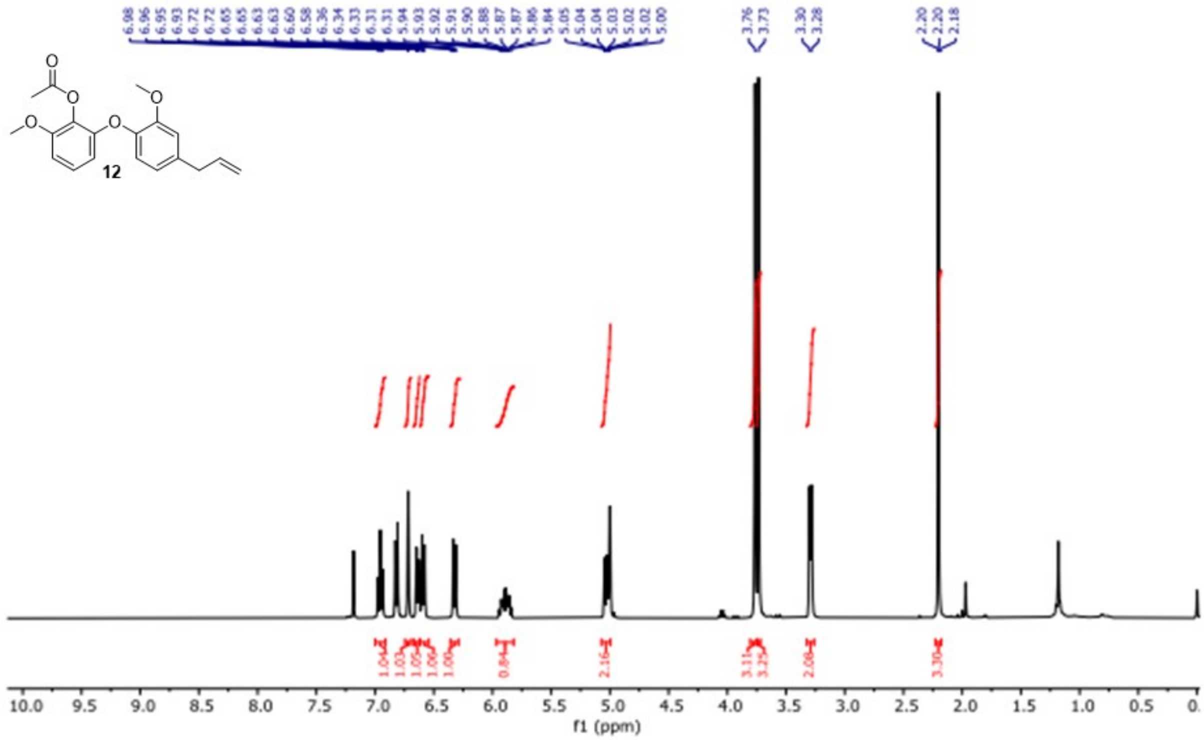
6.98 6.96 6.93 6.72 6.72 6.65 6.65 6.63 6.63 6.60 6.58 6.36 6.34 6.33 6.31 6.31 5.94 5.93 5.92 5.91 5.90 5.88 5.87 5.86 5.84 5.05 5.04 5.04 5.03 5.02 5.02 5.00
3.76 3.73
3.30 3.28
2.20 2.20 2.18
12
1.04 1.03 1.05 1.06 1.00 0.84 2.16 3.11 3.25 2.08 3.30
10.0 9.5 9.0 8.5 8.0 7.5 7.0 6.5 6.0 5.5 5.0 4.5 4.0 3.5 3.0 2.5 2.0 1.5 1.0 0.5 0.
f1 (ppm)

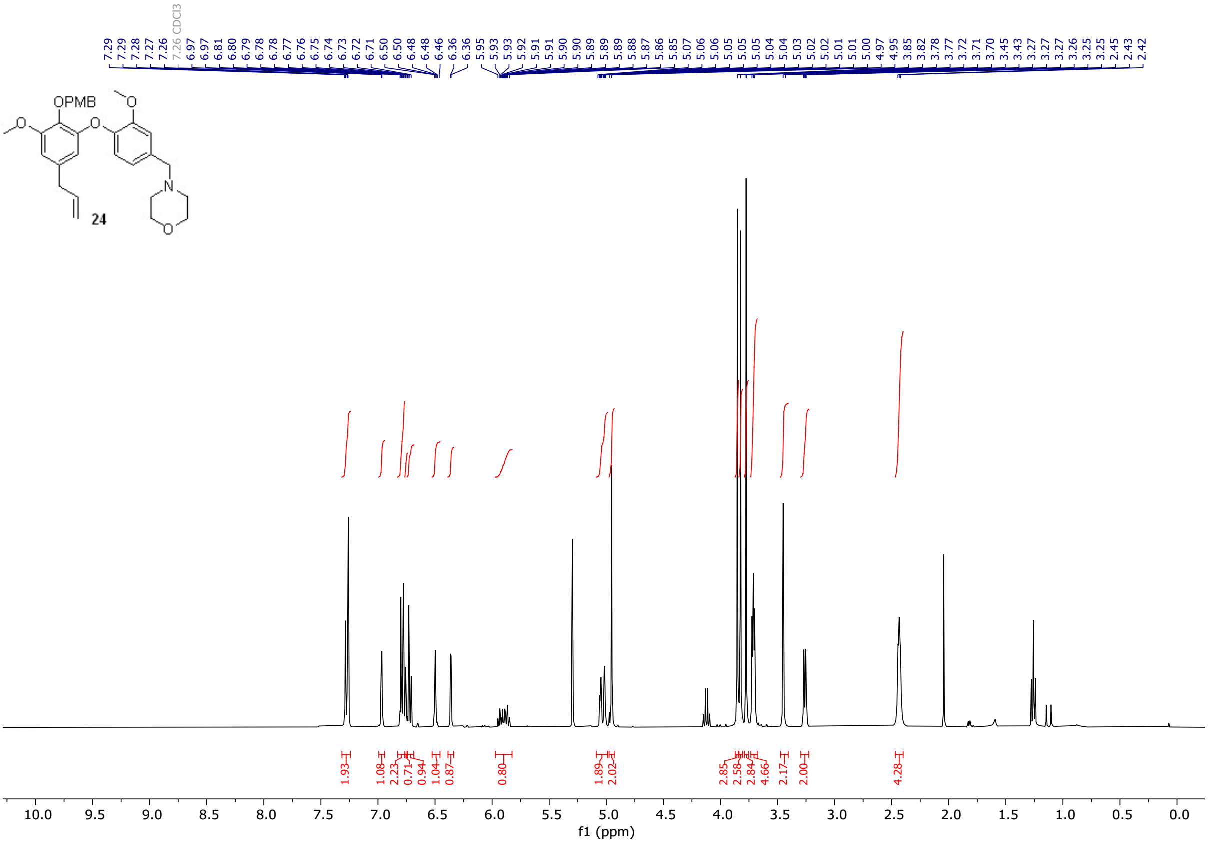
OPMB
24
f1 (ppm)

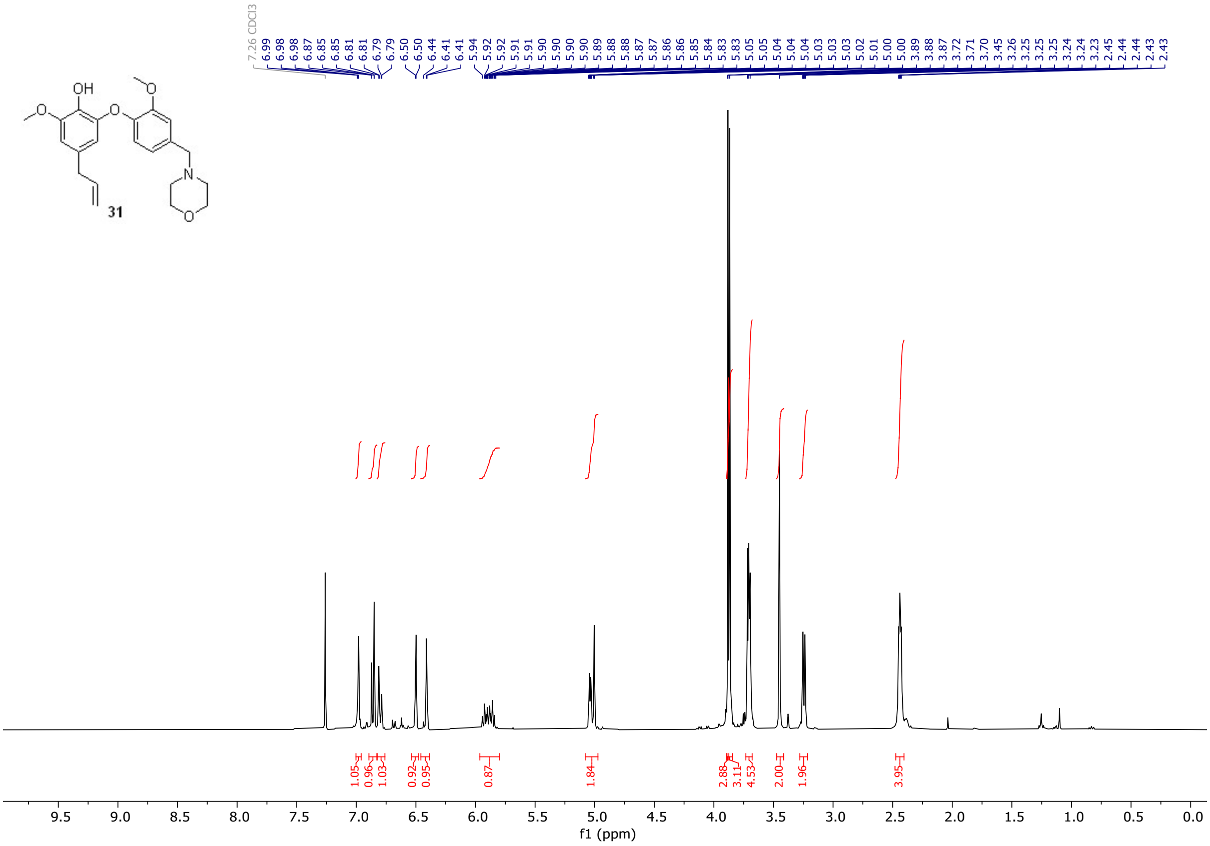
OH
31
f1 (ppm)

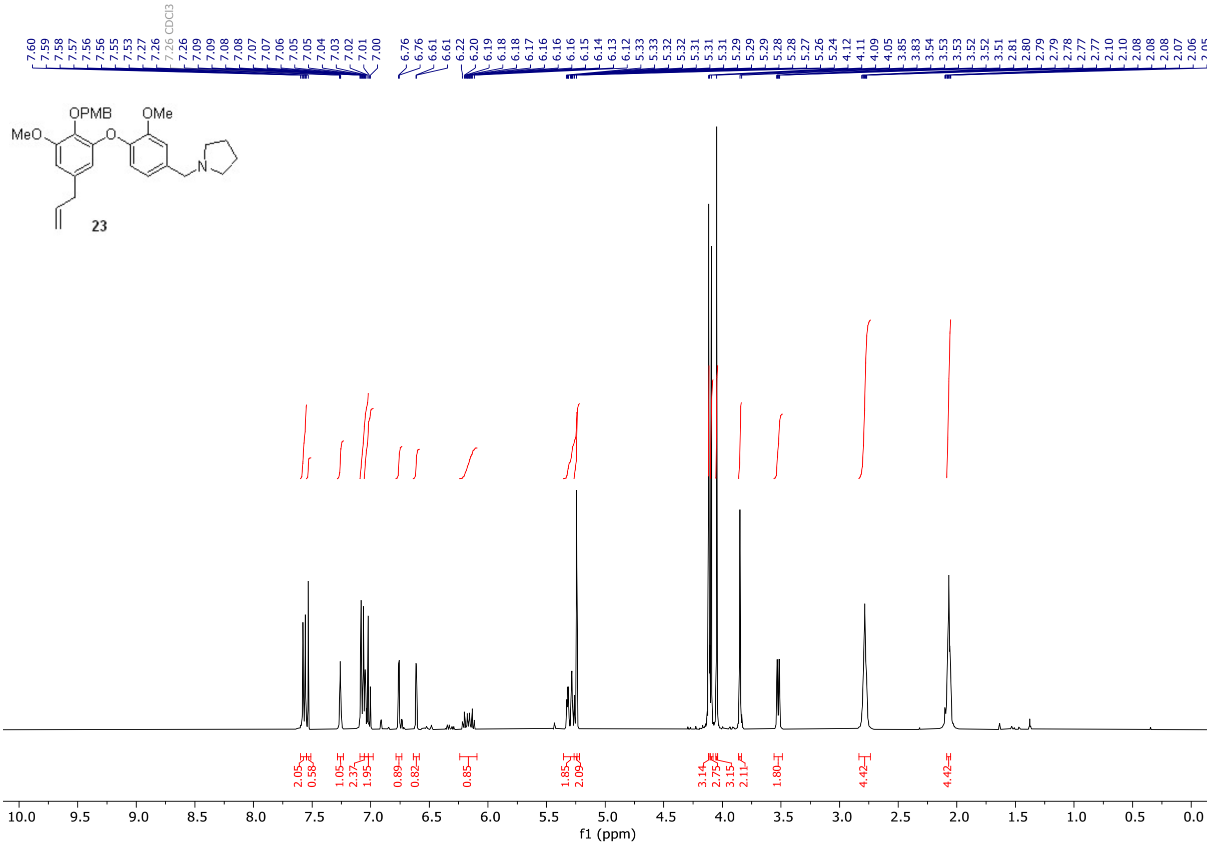

OPMB
OMe
MeO
N
23
f1 (ppm)

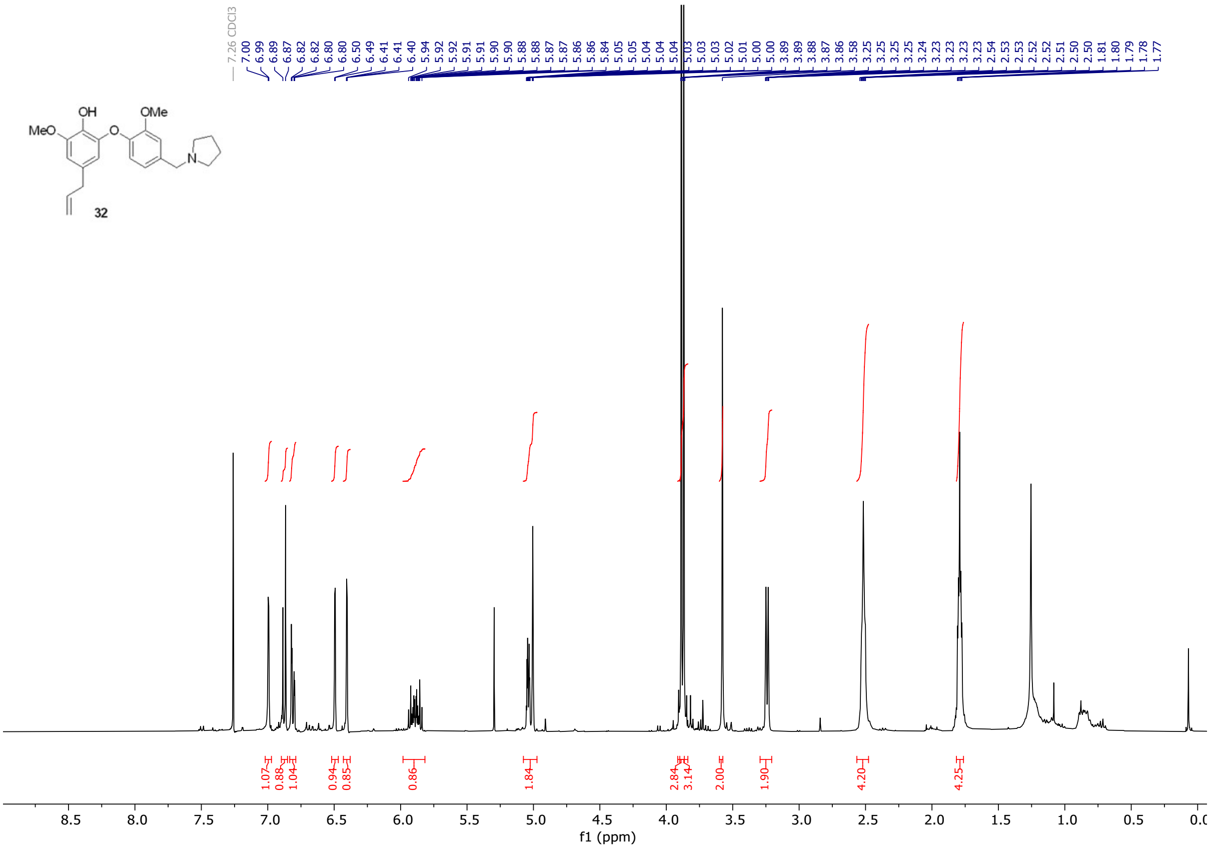

OH
OMe
MeO
N
32
f1 (ppm)

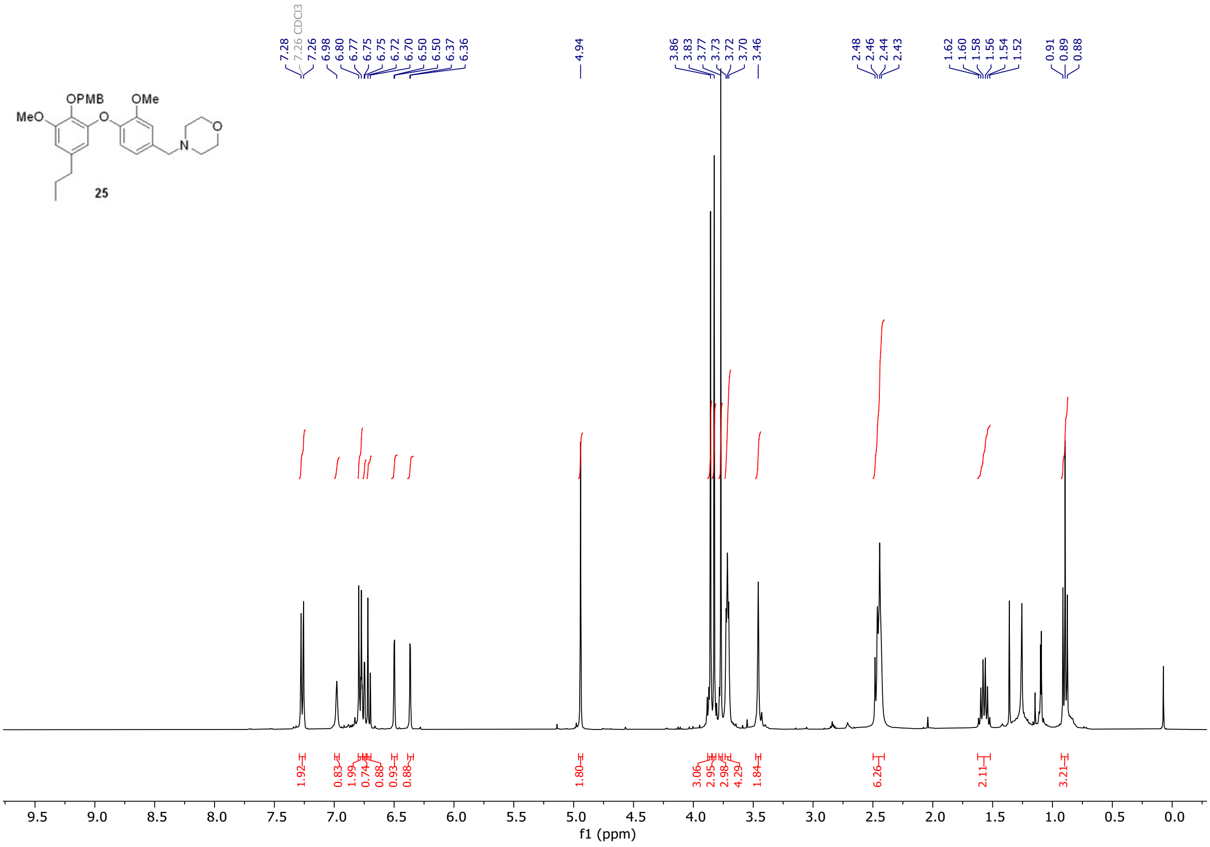

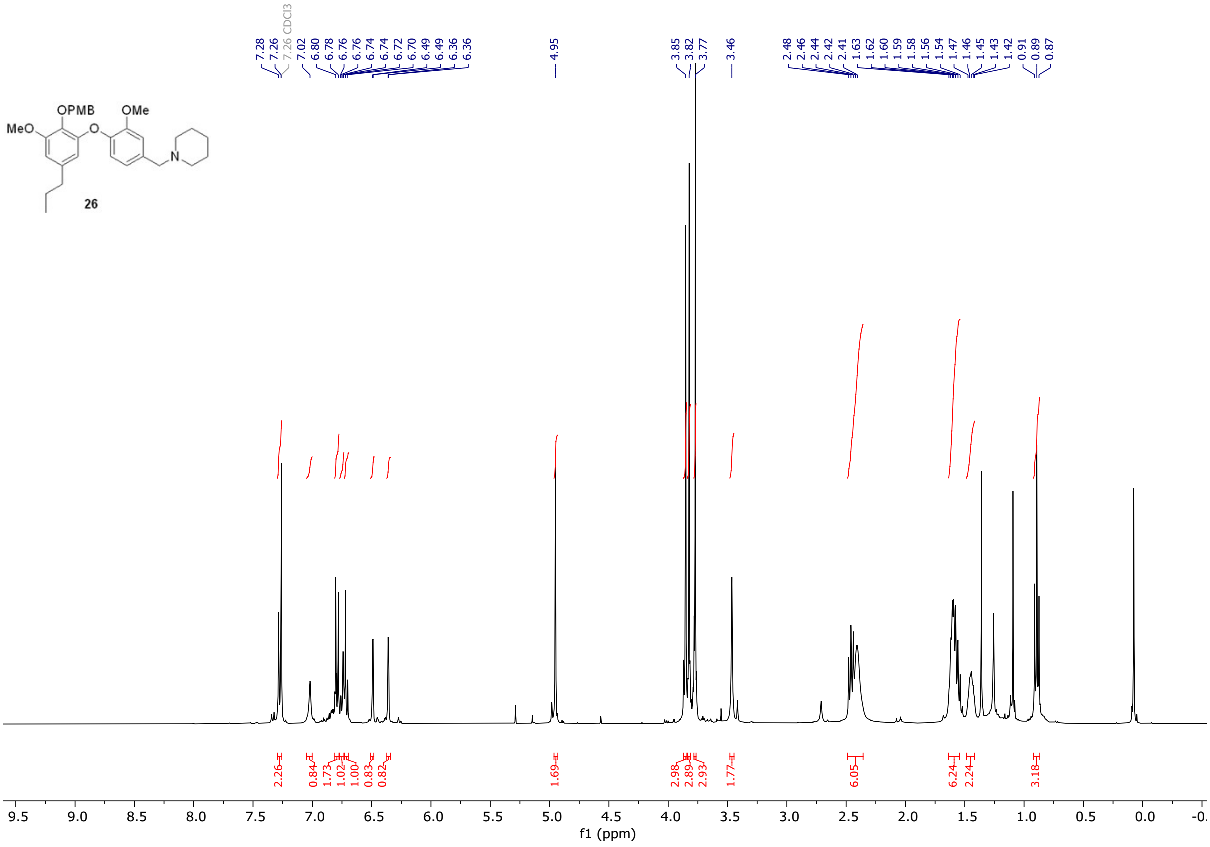

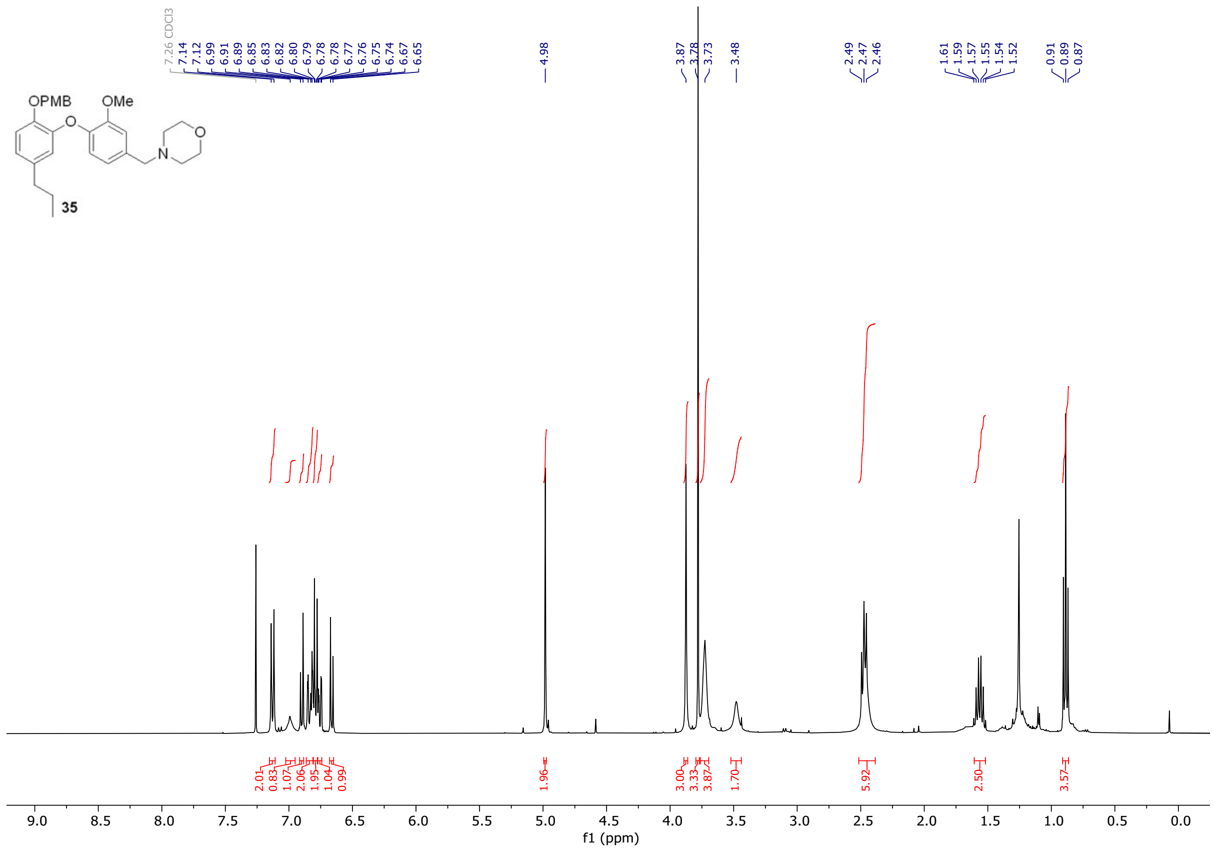

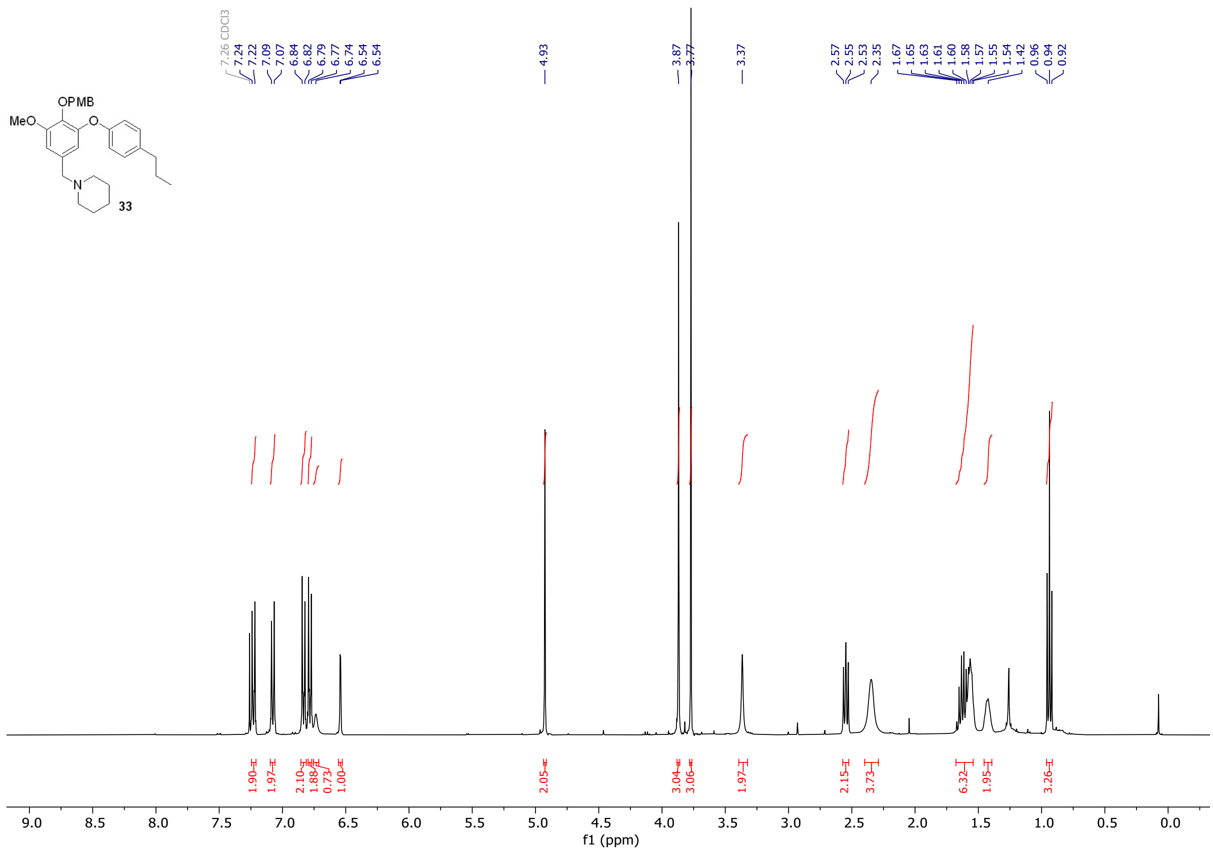

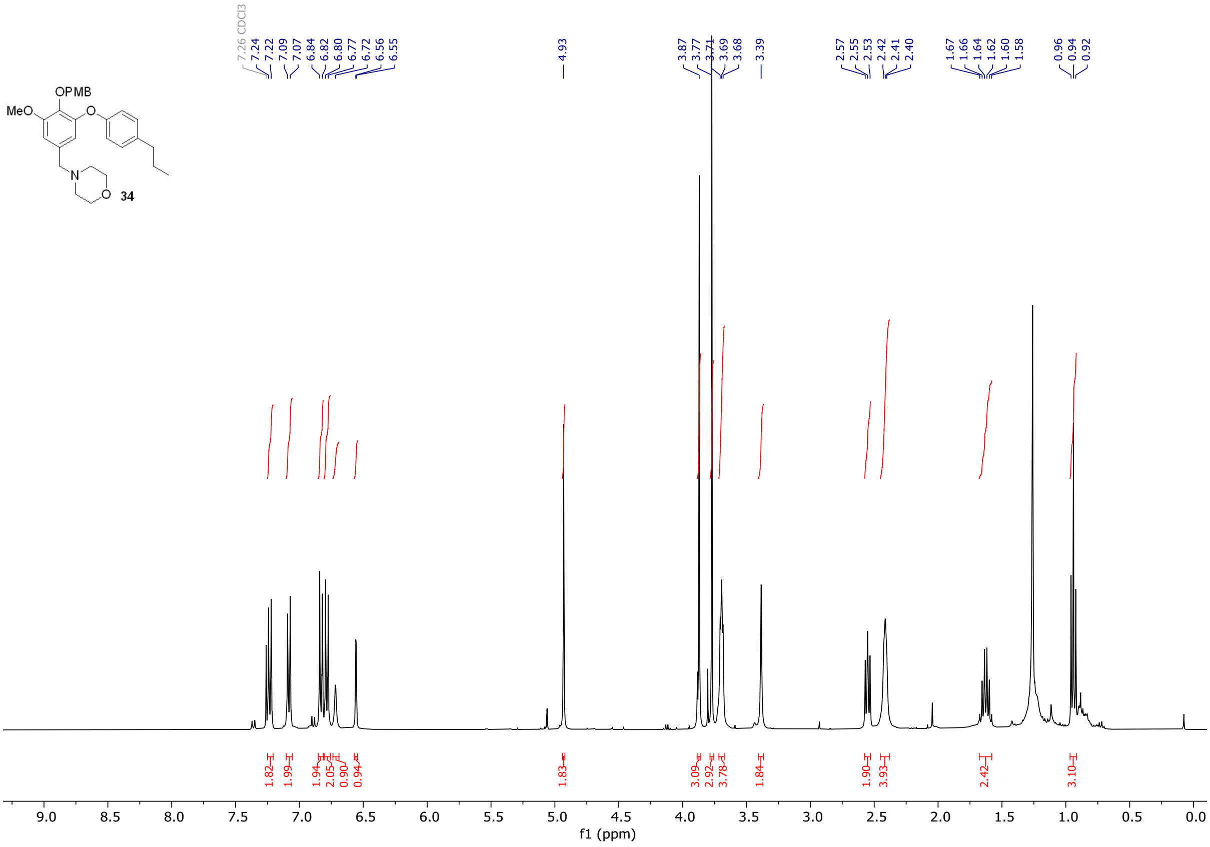
7.26 CDCl3
7.24 7.22 7.09 7.07 6.84 6.82 6.80 6.77 6.72 6.56 6.55
4.93
3.87 3.77 3.71 3.69 3.68
3.39
2.57 2.55 2.53 2.42 2.41 2.40
1.67 1.66 1.64 1.62 1.60 1.58
0.96 0.94 0.92
OPMB
MeO
O
N
O
34
1.82 1.99 1.94 2.05 0.90 0.94 1.83 3.09 2.92 3.78 1.84 1.90 3.93 2.42 3.10
f1 (ppm)

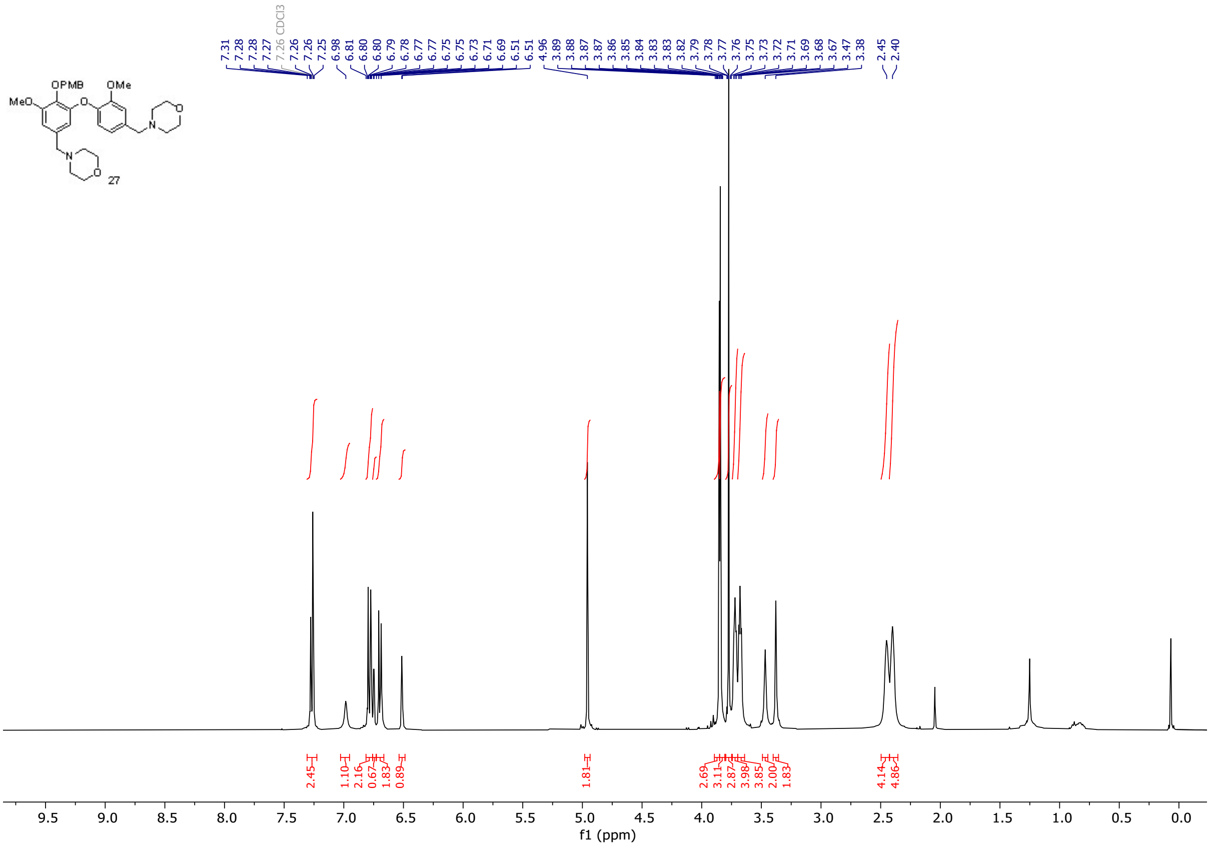
7.31 7.28 7.28 7.27 7.26 CDCl3 7.26 7.25 6.98 6.81 6.80 6.80 6.79 6.78 6.77 6.77 6.75 6.75 6.73 6.71 6.69 6.51 4.96 3.89 3.88 3.87 3.86 3.85 3.84 3.83 3.83 3.82 3.79 3.78 3.77 3.76 3.75 3.73 3.72 3.71 3.69 3.68 3.67 3.47 3.38 2.45 2.40
OPMB
OMe
MeO
O
N
O
27
2.45 1.10 2.16 0.67 1.83 0.89 1.81 2.69 3.11 2.87 3.98 3.85 2.00 1.83 4.14 4.86
f1 (ppm)

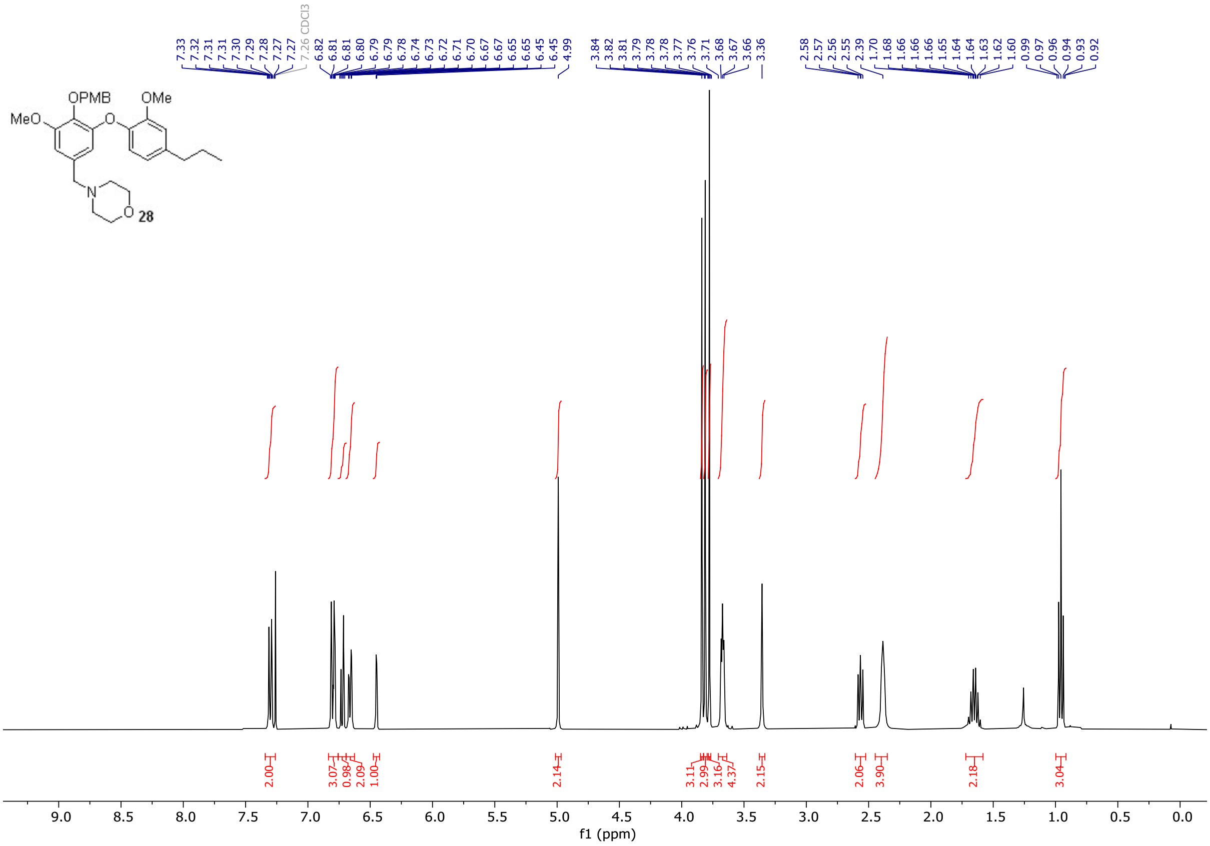

OPMB
OMe
MeO
O
N
O
28
f1 (ppm)

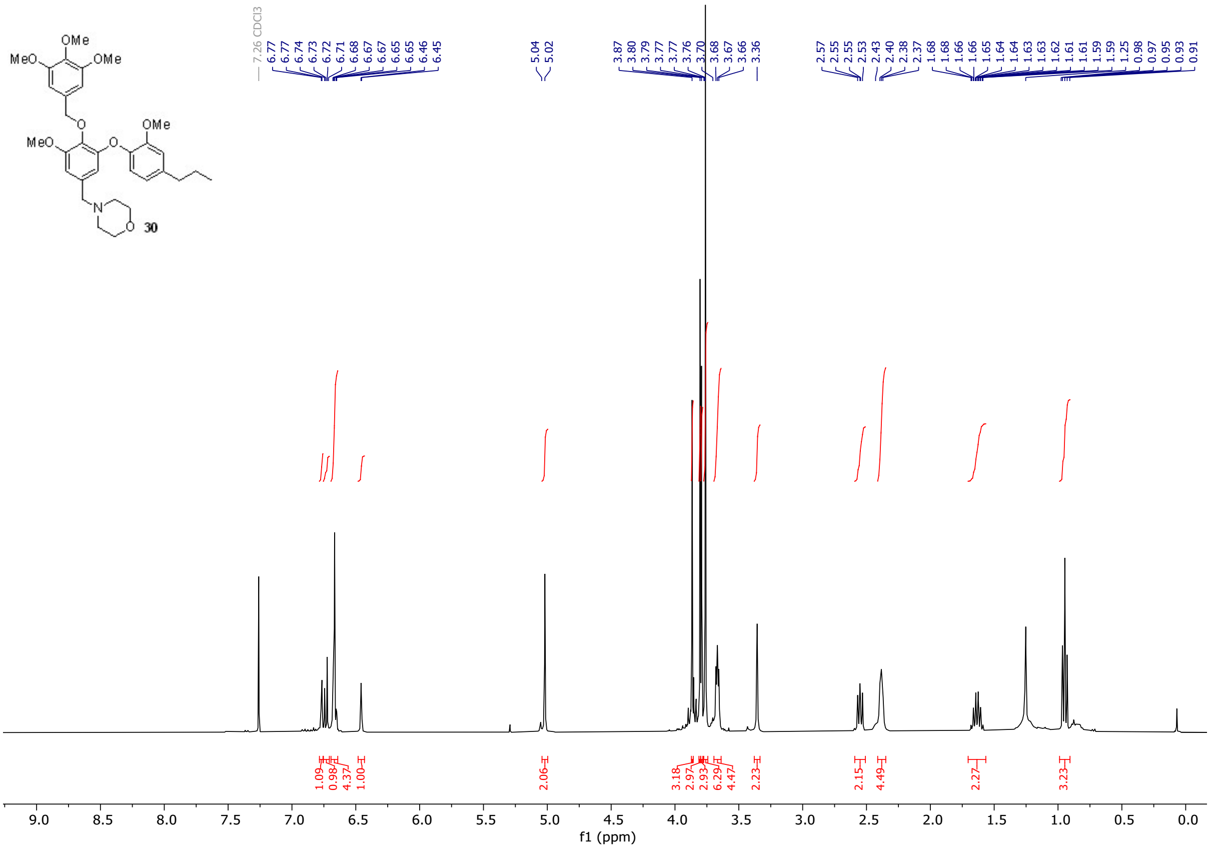

OMe
MeO
OMe
O
MeO
O
OMe
N
O
30
f1 (ppm)

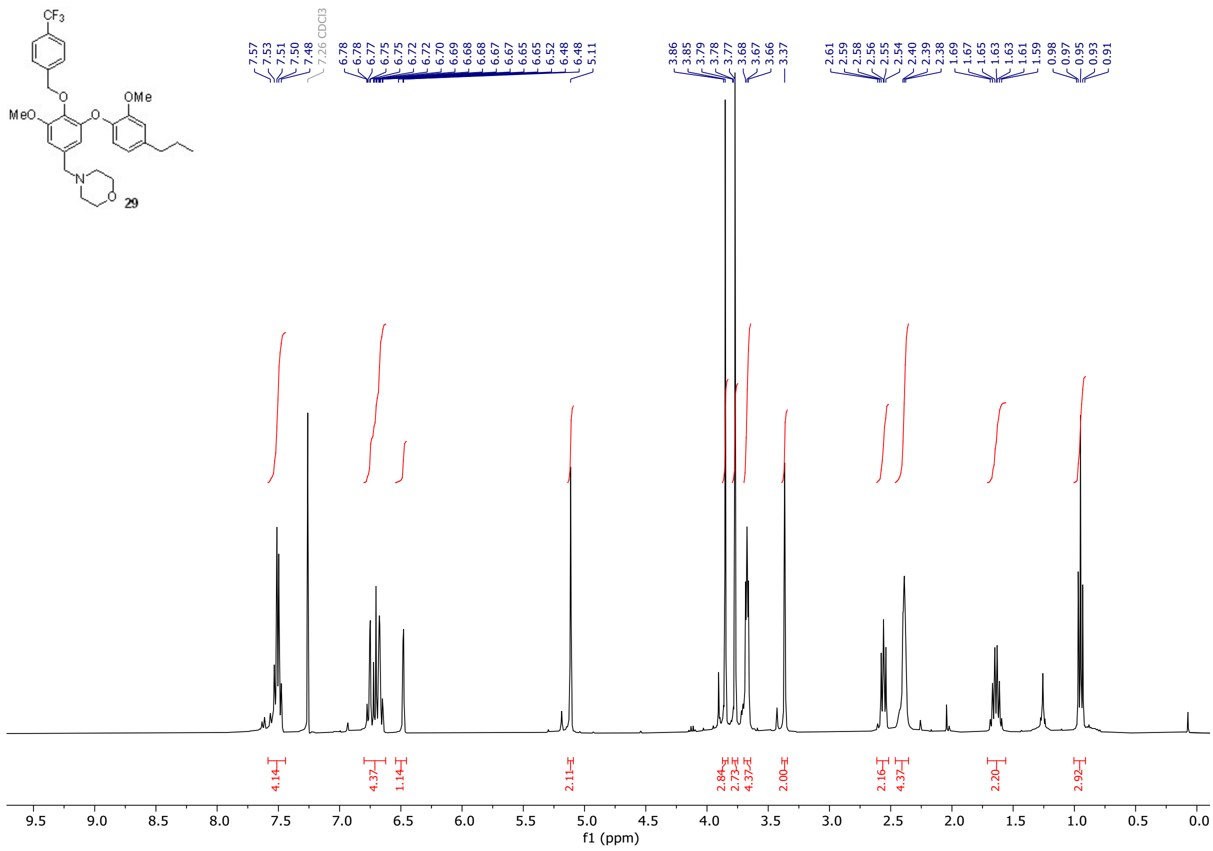

6. References

1. G. Xiong, Z. Wu, J. Yi, L. Fu, Z. Yang, C. Hsieh, M. Yin, X. Zeng, C. Wu, A. Lu, X. Chen, T. Hou and D. Cao, *Nucleic Acids Res.*, 2021, **49**, W5-W14.
2. C. E. Sear, P. Pieper, M. Amaral, M. M. Romanelli, T. A. Costa-Silva, M. M. Haugland, J. A. Tate, J. H. G. Lago, A. G. Tempone and E. A. Anderson, *ACS Infectious Diseases*, 2020, **6**, 2872-2878.
3. R. J. Yoder, Q. Zhuang, J. M. Beck, A. Franjesevic, T. G. Blanton, S. Sillart, T. Secor, L. Guerra, J. D. Brown, C. Reid, C. A. McElroy, Ö. Doğan Ekici, C. S. Callam and C. M. Hadad, *ACS Med. Chem. Lett.*, 2017, **8**, 622-627.
4. R. Nakamura, Y. Obora and Y. Ishii, *Chem. Commun.*, 2008, DOI: 10.1039/B804055A, 3417-3419.
5. 2012.
6. K. V. Sivak, K. I. Stosman, A. A. Muzhikyan, A. G. Alexandrov, N. B. Viktorov, D. D. Vaulina and N. A. Gomzina, *uss. J. Bioorg. Chem.*, 2019, **45**, 425-429.
